# Generation of mixed-valency, modular multispecific antibodies using disulfide-linked Fc–FcγR complexes

**DOI:** 10.1038/s41467-026-72425-5

**Published:** 2026-04-28

**Authors:** Miso Park, Kevin Ly, Bea Parcutela, Hyeran Choi, Carmina Ladra, Asaul Gonzalez, Yead Jewel, Hyunjun Kang, Melissa Valerio, Aparna Krishnan, Timothy W. Synold, Le Xuan Truong Nguyen, Guido Marcucci, John C. Williams

**Affiliations:** 1 Department of Cancer Biology and Molecular Medicine, Beckman Research Institute, City of Hope National Medical Center, Duarte, CA USA; 2 Department of Hematologic Malignancies Translational Science, Gehr Family Center for Leukemia Research, Beckman Research Institute, City of Hope National Medical Center, Duarte, CA USA; 3 Department of Medical Oncology and Therapeutics Research, Beckman Research Institute, City of Hope National Medical Center, Duarte, CA USA; 4 Department of Hematology and Hematopoietic Cell Transplantation, Gehr Family Center for Leukemia Research, City of Hope National Medical Center, Duarte, CA USA

**Keywords:** Antibody therapy, Kinetics, Targeted therapies, Cancer immunotherapy

## Abstract

There is a strong need for multispecific antibodies that possess favorable clinical properties and can be generated through a simple and efficient process. Here, we repurpose the native IgG Fc–FcγR interaction into a universal, covalent docking site using only a single engineered disulfide bond, providing a simple solution to bypass the complex, bespoke engineering typically required for multispecific antibody design. The resulting Fc–FcγR complex forms a homodimeric Fc with one ligand, with the FcγR offering both N- and C-termini for independent functionalization, enabling single- or dual-payload formats, including masked designs for conditional activation. Introducing the disulfide between Fc (A330C) and FcγRIIIa (I106C) yields stable, covalently linked complexes that do not require post-expression modification, are compatible with standard mammalian expression, and support payloads such as anti-CD3, anti-CD28, IL-2, and protease-activated IL-2. These compounds exhibit potent, antigen-selective cytotoxicity in vitro and in vivo, with tunable avidity and reduced off-target activity. This plug-and-play platform overcomes key developability bottlenecks and enables rapid, scalable creation of next-generation antibody therapeutics.

## Abstract (web summary)

The manufacture of multispecific antibodies can require complex engineering. Here, the authors develop Fc-FcγR disulfide-linked complexes that can be used for modular delivery of diverse payloads.

## Introduction

Due to their high specificity and favorable pharmacological properties, significant efforts have been made to engineer monoclonal antibodies (mAbs) to enhance their therapeutic properties or equip them with novel functionality^1^. Of these efforts, bispecific and multispecific mAbs that engage immune cells (T-cell, NK cell, etc.) have yielded significant clinical results, especially in hematological malignancies^2^. A major challenge in the development of bispecific antibodies, such as αCD3 T-cell engagers (TCEs), is optimizing biological potency with pharmacology and manufacturability. The immunoglobulin G (IgG) scaffold contains two heavy and two light chains; to build a TCE one antigen-binding fragment (Fab) must bind the tumor antigen and the other CD3. Even when all four chains are expressed equally, stochastic pairing produces twelve IgG variants, and only one has the intended dual specificity. The resulting 1/12 theoretical yield, together with the need to eliminate these mis-paired species (e.g., one species that is bivalent to CD3 can activate T cells nonspecifically) has prompted extensive engineering to enforce correct pairing and improve production efficiency^3–7^.

Two broad approaches have emerged. One tethers single-chain variable fragments (scFvs) in tandem with peptide linkers^8^. The other keeps an intact fragment crystallizable region (Fc) of mAb to extend half-life but drives heterodimer formation through knob-into-hole mutations^9,10^, electrostatic steering^11^ or Fab-arm exchange^12^. Additional refinements include CrossMab junctions^13^, grafting T-cell receptor constant domains^14^, and adopting common light chains^15^ to further ensure proper assembly.

Moreover, initial bispecific designs using these technologies were monovalent to each intended target (e.g., monovalent to CD3 and the tumor-associated antigen {TAA}). While this design is sufficient for strictly unique tumor antigens, many TAAs are also expressed on healthy tissues albeit at much lower levels. The loss of avidity to the target requires a higher affinity of the tumor targeting Fab and thus increases on-target, off-tissue toxicities. To address this possibility, additional antigen targeting domains are fused to the protein to reintroduce avidity. This includes incorporation of tandem scFvs, mixed use of common light chains, etc^16^. While promising results are being observed in preclinical and on-going clinical trials, these methods also require extensive engineering and optimization. Modular systems such as SpyTag/SpyCatcher, sortase-mediated ligation, and split-intein approaches offer alternatives, however, they often require post-expression modification, non-natural components, or are limited in stoichiometric control^17^. Despite these advances, a generalizable, modular strategy to rapidly generate and screen mixed-valent, multispecific antibody-based biologics remains elusive.

Beyond binding to the intended targets (e.g., TAA and CD3 for a TCE), additional engineering in the Fc region including point mutations in the Fc domain are also often incorporated in IgG-based approaches to inhibit Fcγ receptors (FcγR) I–III binding, improve PK (pharmacokinetics) by reducing liver uptake and eliminate antibody-dependent cell-mediated cytotoxicity (ADCC). These mutations include LALA^18^, STR^19^, among others. Alternatively, some designs incorporate the low affinity IgG4 Fc, but require the mutation, S228P, to reduce Fab arm exchange^20^. Retaining the Fc portion in multispecific antibodies preserves neonatal Fc receptor (FcRn) engagement and its pharmacokinetic benefit as the FcRn binding site on the Fc is separate from the FcγR interface.

In light of these constraints, we sought to simplify the approach to generating bispecific antibody-based biologics. First, we recognized that the FcγR extracellular regions specifically bind to the Fc region of an IgG1 naturally and with relatively high affinity (~1 nM to 1 μM)^21^. Given this native interaction, we searched the Protein Data Bank (PDB) for structures of the Fc-FcγR to identify residues at the interface that would support the formation of an intermolecular disulfide bond. Among different candidates, we identified residues at position 330 in the Fc (following EU numbering on human IgG1 Fc) and 106 in the FcγRIII (UniProt ID P08637) as a potential pair. Second, we understood that we could fuse scFvs or cytokines at the N- or C- termini of FcγR extracellular domain to add a new functionality (e.g., generating bispecific antibodies). We also understood that this approach affords a mixed valent TCE. Although an IgG is dimeric, its Fc engages only one FcγR in an asymmetric 2:1 complex^22^. When the engineered disulfide between Fc A330C and FcγRIII I106C forms, the molecule retains bivalent Fab binding to the first target and presents a single FcγR fusion for the second. Also, as the fusion already occupies the FcγR binding pocket, no additional Fc mutations are required to silence interactions with FcγR classes I–III.

**Fig. 1:**
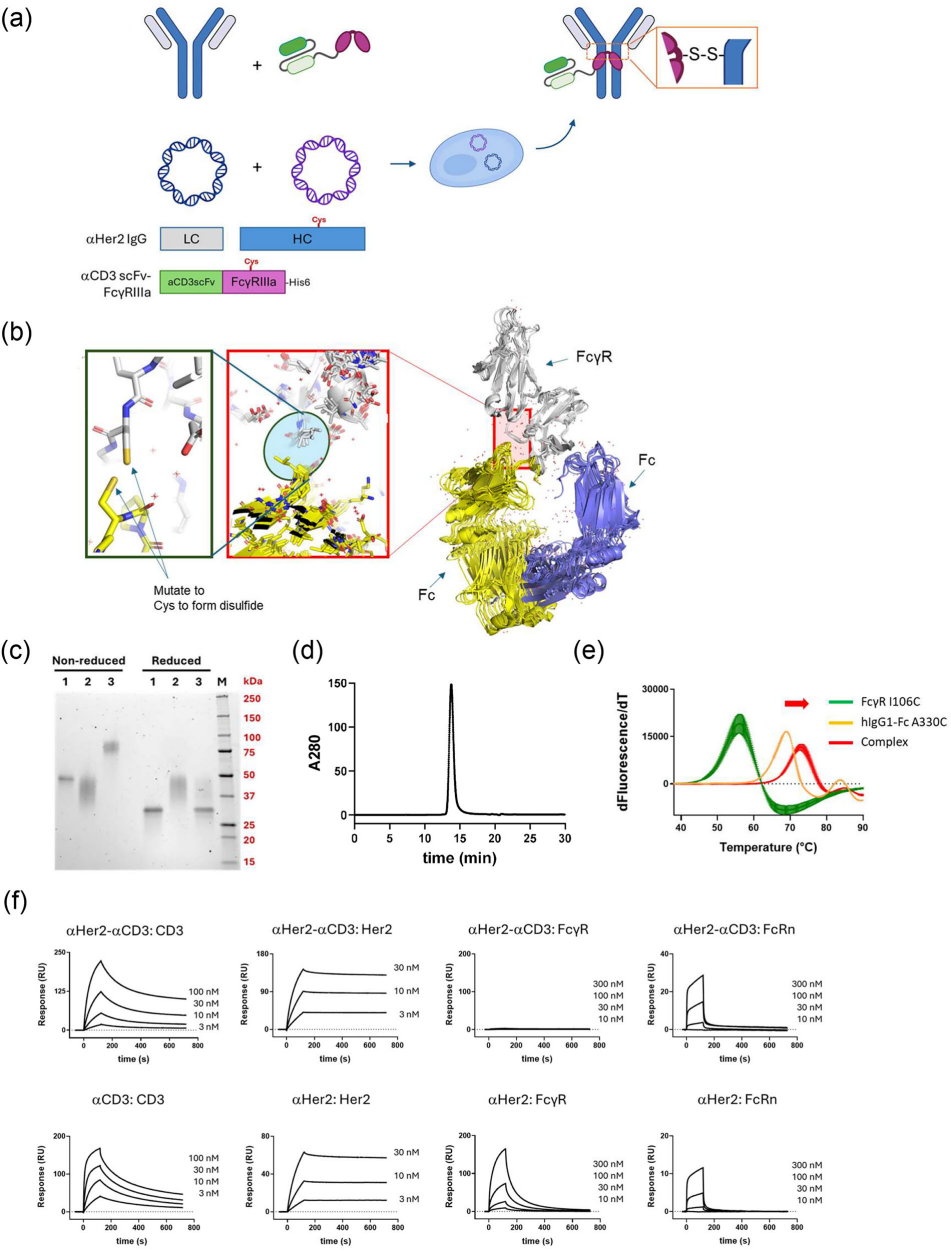
Antibody functionalization through disulfide linked Fc-FcγR fusions. **a** Schematic of antibody functionalization through disulfide-linked Fc-FcγR fusions. Mammalian expression vectors encoding IgG and FcγR fusions are co-transfected into a single mammalian cell line. Created in BioRender. Park, M. (2026) https://BioRender.com/egv49dj. **b** Superposition of Fc and FcγR crystal structures from the Protein Data Bank (PDB entries: 1T89, 6EAQ, 5XJE, 5YC5, 7URU, 5XJF, 5D6D, 3WN5), aligned via the Fc-FcγR interface. This analysis reveals two residues—one from the Fc and one from the FcγR—ideally positioned for disulfide formation, with a distance of 2.1 Å between the sulfur atoms of each cysteine residue. PyMOL Molecular Graphics System (version 2.5.5, Schrödinger, Inc.) was used for graphical illustrations. **c** SDS-PAGE analysis of the purified Fc-FcγR complex. Lane 1: Fc A330C, Lane 2: FcγR I106C, Lane 3: Fc-FcγR complex, Lane M: Protein ladder. The diffuse FcγR I106C band is consistent with glycosylation which produces the expected range of glycoforms on SDS–PAGE. This analysis was repeated independently three times with similar results. **d** HPLC-SEC chromatogram of the Fc–FcγR complex collected under native conditions, demonstrating a single, well-defined species. **e** Protein thermal shift assay. The melting temperature (T_M_) is derived from the thermal melt curve using a protein-binding dye. The disulfide-linked Fc-FcγR complex (red trace) exhibits a notable increase in T_M_ compared to its individual components, indicating enhanced thermal stability upon complex formation. Data are presented as mean values +/− SD from three technical replicates. **f** Surface plasmon resonance (SPR) analysis of target binding. CD3, Her2, FcγRIIIa, or FcRn were individually immobilized on SPR sensor chips, and increasing concentrations of the αHer2–αCD3 complex or the corresponding parental antibodies were injected over each surface. Response units were recorded and analyzed using Biacore T200 Evaluation Software.

Herein, we demonstrate that this is a viable approach to functionalize IgGs. We first show the co-expression, compatible with standard mammalian expression systems, of the extracellular regions of FcγRIII and IgG bearing the cysteine substitution in the same cell are secreted and can be purified as an intact complex (Fig. 1a). Importantly, this approach does not require post-expression chemical modification or complex purification workflows. We further show the complex is stable in mouse serum at 37 °C over 7 days. Next, we fused anti-CD3 scFvs to the N-, C- or both termini of the first two domains of FcγRIII I106C and expressed this with multiple IgGs bearing the A330C mutation to create distinct TCEs. We further characterized these TCEs in vitro and in vivo, showing these constructs achieve tumor killing across multiple tumor cell types with high fM - low pM EC_50_ and drive robust cytokine production. We also show that we can add other functionalities to the FcγR domains including anti-CD28 scFvs, IL2 and tumor-activated (masked) IL2. Collectively, this “templated” disulfide approach allows the rapid generation and screen mixed-valent, multiple-specific, antibody-based biologics.

## Results

### Establishing the intermolecular disulfide bond

Analyzing multiple crystal structures of Fc–FcγR (I–III) complexes deposited in the PDB, we identified several residue pairs—one from the Fc and one from the FcγR—that could support the formation of an intermolecular disulfide (Fig. 1b). We initially focused on Leu330 in the Fc and Ile106 in FcγR from PDB ID5D6D, where the β-carbon distance is 5.5 Å. Rotamer selection using PyMOL indicated that mutating each residue to cysteine would support a favorable disulfide geometry. The corresponding residues in human IgG1 Fc, Ala330, and the soluble fragment of FcγRIIIa, Ile106, were mutated, expressed individually and together in ExpiCHO cells, and purified using affinity and size exclusion chromatography (SEC). Both proteins were expressed at high levels and remained stable throughout purification.

To assess disulfide formation, we analyzed the purified proteins by reducing and non-reducing SDS-PAGE (Fig. 1c). A clear shift was observed in the non-reducing gel only when both cysteine-containing molecules were co-expressed, consistent with the formation of an intermolecular disulfide. Densitometric analysis of non-reducing SDS-PAGE indicated >90% of the FcγRIIIa I106C co-purified with Fc A330C as a disulfide-linked complex. Control samples lacking the engineered cysteines did not show this shift (Supplementary Fig. 1a). Fc–FcγR complex was run on HPLC (high-performance liquid chromatography)-SEC in native condition. A single symmetric peak was observed, supporting a pure and homogeneous stable complex (Fig. 1d). We further verified disulfide formation using differential scanning fluorimetry (DSF). The melting temperature (T_M_) of FcγRIIIa I106C was 54.9 °C (±0.3 °C, triplicates from N of 2), while IgG1 Fc A330C melted at 67.6 °C (±0.2 °C, triplicates from N of 2). The disulfide-linked complex exhibited a single transition at 72.0 °C (±0.2 °C, triplicates from N of 2) (Fig. 1e), consistent with a stabilized, covalently tethered complex.

To test whether this strategy was compatible with Fc variants used in therapeutic settings, we introduced the S239D and I332E mutations—known to enhance FcγRIII binding and ADCC—into the Fc A330C background. Co-expression with FcγRIIIa I106C again yielded a disulfide-linked complex, as evidenced by a shift in non-reducing SDS-PAGE (Supplementary Fig. 1b). Notably, these affinity-enhancing mutations reduced the thermal stability of the Fc alone (T_M_ = 50.6 ± 0.1 °C, triplicates from N of 2), but the disulfide-linked complex remained stable, with a T_M_ of 70.1 °C ( ± 0.2 °C, triplicates from N of 2) (Supplementary Fig. 1c).

Finally, to assess the stability of the complex under physiologically relevant conditions, we incubated the purified disulfide-linked complex in PBS and in mouse serum at 37 °C for 7 days. SDS-PAGE and Western blot (WB) analysis of samples collected over time confirmed that the complex remained intact in both conditions (Supplementary Fig. 1d). Additionally, the stability of the complex with human serum was investigated by Western Blot analysis. Only trace (<1%) higher–molecular-weight species were detected over time, consistent with minor self-association rather than albumin exchange, and the complex remained predominantly intact throughout the 7-day incubation (Supplementary Fig. 1e).

Critically, when we introduced mutations into the Fc that reduce FcγRIIIa binding—while retaining the cysteine at position 330—and co-expressed this construct with the cysteine-mutated FcγRIIIa soluble domain, we failed to produce the complex despite robust expression of both components, indicating that the native interaction is essential for guiding and stabilizing the intermolecular disulfide (Supplementary Fig. 2a). Additionally, we evaluated FcγRI as a potential partner for assembling Fc–FcγR complexes, given its high affinity for the Fc region. While initial results suggest that this approach is feasible, FcγRI fusion constructs exhibited suboptimal expression and reduced assembly efficiency when combined with anti-CD3 or other payloads. These findings indicate that further engineering will be required to achieve suitable manufacturability and stability. Accordingly, the present study focuses on FcγRIIIa-based constructs.

### Functionalization of IgGs

Given these results, we turned to FcγR fusions and full IgGs. Here, we added the scFv of the αCD3 antibody (patent US9650446B2) to the N-terminus of FcγRIIIa and used the αHer2 antibody, trastuzumab, as a representative IgG1. As before, co-expression of the FcγRIIIa and IgG (both heavy and light chains) with cysteines encoded at the respective positions produced the complex, as judged by the presence of a single band on non-reducing SDS-PAGE (Supplementary Fig. 2b). A reducing SDS-PAGE gel of the complex produced three bands, each consistent with the masses expected for the individual components.

Next, we used Surface Plasmon Resonance (SPR) to characterize the effect of the intermolecular disulfide bond on antigen binding. First, we observed comparable binding of the complex and the αCD3 scFv to immobilized CD3 (single chain of CD3ε/γ) (Fig. 1f). We also observed comparable binding to immobilized Her2 relative to the parental IgG, albeit avidity effects of the “IgG” are present and do not accurately report on the “monovalent” interaction. Conversely, to assess whether the FcγRIIIa binding site on the Fc was blocked in the complex, we immobilized FcγRIIIa on the SPR chip and passed both the complex and the parental IgG over the surface. A substantial reduction in signal was observed for the complex, consistent with occlusion of the FcγRIIIa binding site, whereas the parental antibody bound with an affinity consistent with the literature (K_D_ of 190 ± 30 nM, N of 3) (Fig. 1f)^23^. While the designed complex successfully blocks FcγR interactions, it retains binding to FcRn as measured by SPR, comparable to the parental antibody (Fig. 1f), indicating that desirable pharmacokinetic properties are preserved.

**Fig. 2:**
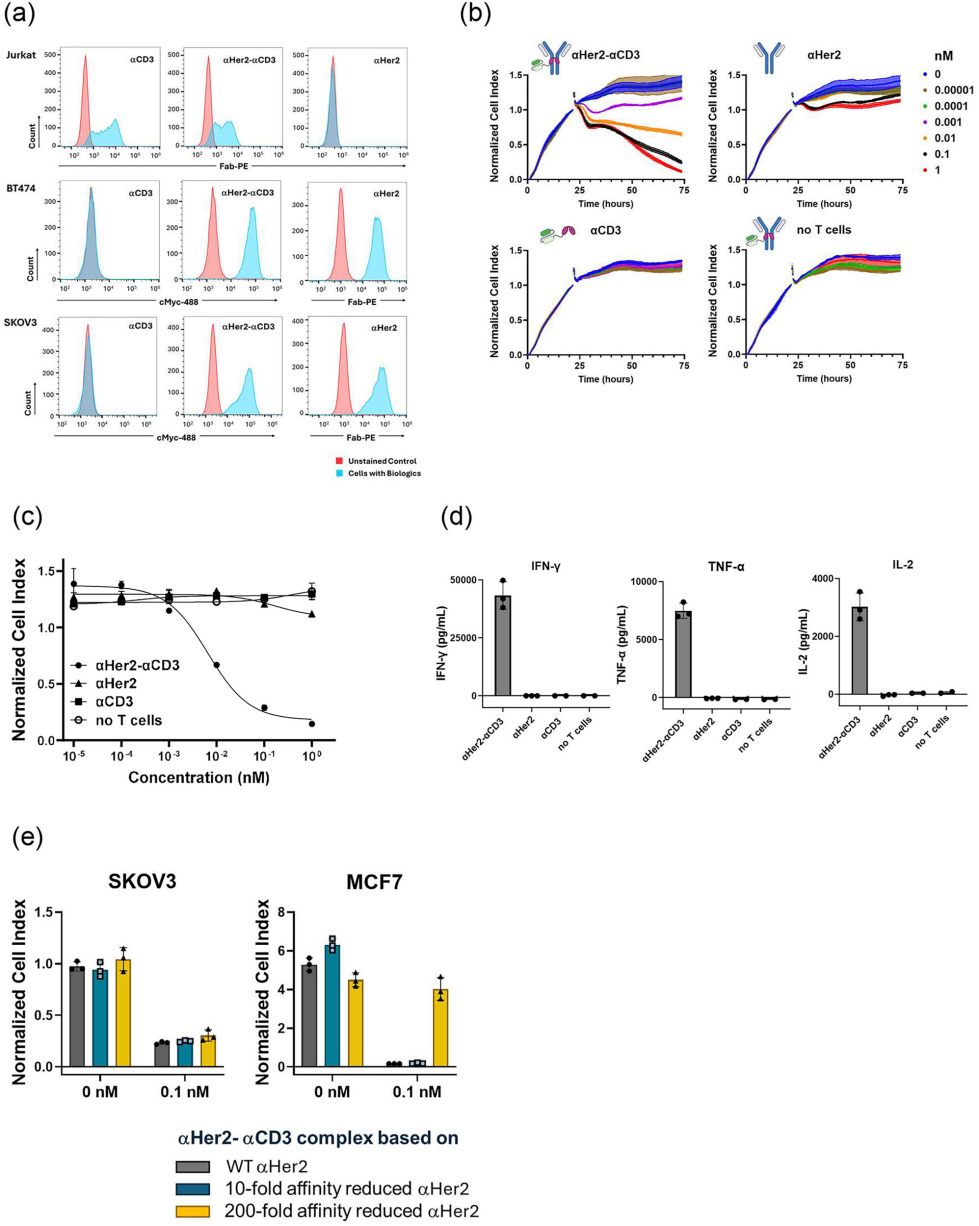
In vitro activity of a disulfide-linked bispecific Her2-T cell engager. **a** Cell binding of the αHer2-αCD3 complex assessed by flow cytometry. Fluorescence histograms were generated for Jurkat (CD3-positive), BT474 (Her2-positive), and SKOV3 (Her2-positive) cells. Non-stained cells (red trace) and biologic-treated cells (blue trace) are shown. Fab-PE indicates anti-human Fab antibody conjugated to PE; cMyc-488 indicates anti-cMyc antibody conjugated to Alexa488. The αHer2-αCD3 complex shows comparable cell-binding affinity to the parental antibodies. **b** Real-time analysis of SKOV3 cell killing mediated by the αHer2-αCD3 complex. Human T cells were added at an effector-to-target (E:T) ratio of 10:1, and biologics were added at concentrations ranging from 0.00001 to 1 nM. Cell impedance was continuously monitored and normalized at the time of treatment. Concentration-dependent cell killing was specifically observed for the αHer2-αCD3 complex in the presence of T cells, whereas parental antibodies and the complex without T cells showed no significant cytotoxicity. Data are presented as mean values +/− SD from three technical replicates for the samples with T cells and from two technical replicates for no T cells. Inserted figures are created in BioRender. Park, M. (2026) https://BioRender.com/s39wq27. **c** Normalized cell index at 48 h post-treatment. Samples include: αHer2-αCD3 (closed circle), αHer2 alone (closed triangle), αCD3 alone (closed square), and αHer2-αCD3 complex without T cells (open circle). Data are presented as mean values +/− SD from three technical replicates for αHer2-αCD3 and αHer2, and from two technical replicates for αCD3 and no T cells. **d** Cytokine production (IFN-γ, TNF-α, and IL-2) measured 48 h after treatment with 1 nM of each biologic and T cells at a 10:1 E:T ratio. Data are presented as mean values +/− SD from three technical replicates for the samples with T cells and from two technical replicates for no T cells. **e** SKOV3 and MCF7 cell killing assays using affinity-reduced αHer2-αCD3 complexes with human T cells at a 10:1 E:T ratio. Normalized cell index at 48 h post-treatment is plotted for untreated (0 nM) and 0.1 nM treated groups: αHer2-αCD3 (black), αHer2mB-αCD3 (Y92A mutation, blue) and αHer2mA-αCD3 (H91A mutation, yellow). Data are presented as mean values +/− SD from three technical replicates.

To characterize the effect of the disulfide bond on the ability of each component of the complex to bind to target cells, we conducted a series of analytical cytometry studies. First, we used Jurkat cells to assess binding to CD3 and observed comparable binding to the αCD3 Fab alone (Fig. 2a). Next, we assessed Her2 binding using Her2-overexpressing cell lines BT474 and SKOV3. In this case, we used an antibody to detect a cMyc tag on the αCD3 scFv–FcγRIIIa fusion. In both cell lines, the disulfide-bridged complex bound to the cells at levels comparable to the parental antibody (Fig. 2a). To rule out unintended or non-specific interactions arising from the novel complex, we generated and characterized additional constructs. First, the Her2 disulfide complex was generated using FcγRIIIa without the CD3 scFv fusion (αHer2–αCD3null). No binding to Jurkat cells was observed (Supplementary Fig. 2c). Likewise, αCD3–FcγRIIIa only without Her2 IgG was incubated with BT474 and SKOV3 cells, and again, no binding was observed (Fig. 2a).

Given these results, we turned to in vitro T cell activity assays to characterize the potency of the T cell bispecific. BT474 cells were plated, and the αHer2–αCD3 complex was titrated in the presence of Jurkat cells engineered with an NFAT-luciferase reporter as a surrogate for TCR activation. Using luminescence as a readout, we observed potent activation of the NFAT pathway with an EC₅₀ of ~8 pM (N of 1, supplementary Fig. 2d). No activity was observed from the αCD3 scFv–FcγRIIIa fusion alone or in the absence of target cells. Likewise, only minor activation was observed at much higher concentrations using the parental antibody (i.e., lacking FcγRIIIa fusion). Similar results were observed using SKOV3 as target cells (Supplementary Fig. 2d).

We next turned to primary T cells from healthy donors. In these experiments, we used real-time cell analysis based on impedance^24^. SKOV3 cells were plated and grown to near confluency, and T cells isolated from a healthy donor (M46, 46-year-old male) were added at an effector-to-target (E:T) ratio of 10:1. The αHer2–αCD3 complex was titrated, and impedance was monitored over 72 h (Fig. 2b). As shown, cell killing was dose dependent. At 48 h post-treatment, an EC₅₀ of 8.1 pM ( ± 2.3 pM) was calculated (Fig. 2c). The EC_50_ value was derived from technical triplicates, and standard deviation reflects two individual experiments conducted on separate dates using freshly isolated T cell isolation from the same donor’s frozen PBMCs. Critically, the parental antibody titrated over the same range failed to induce tumor cell killing. The αCD3–FcγRIIIa fusion also failed to kill tumor cells at concentrations equivalent to the complex Supplementary Movie 1. In addition to tumor killing, we quantified IFN-γ, TNF-α, and IL-2 levels across treatments at 1 nM and 48 h (Fig. 2d). Consistent with potent T cell activation, only the αHer2–αCD3 complex led to significant IFN-γ production. Elevated levels of TNF-α and IL-2 were also observed (Fig. 2d).

To ensure robustness and capture true biological variability, we also assessed T cell-mediated cytotoxicity using T cells isolated from PBMCs of eight independent donors. Seven of eight donor samples exhibited comparable BT474 cell killing at 0.1 nM of αHer2–αCD3, while one donor PBMC showed no detectable cytotoxic activity under the same condition (Supplementary Fig. 2e). This lack of response could be attributed to donor-specific factors such as lower baseline T cell activation or reduced CD3 level. Together, these data demonstrate that the disulfide-linked complex retains dual specificity and drives potent T cell activation and tumor cell killing in vitro.

To demonstrate the advantages of bivalent target binding through the full IgG format for enhancing avidity and selectivity, compared to most clinically approved bispecific T cell engagers that rely on monovalent binding, we introduced mutations (H91A and Y92A) in the complementarity-determining regions of αHer2 to generate αHer2 variants with reduced target binding affinity. H91A in the light chain reduces the affinity by ~200-fold and Y92A in the light chain reduces the affinity by ~10-fold^25^ (Supplementary Fig. 2f). Both αHer2mA-αCD3 (αHer2 H91A) and αHer2mB-αCD3 (αHer2 Y92A) exhibited low cell index values in a high Her2-expressing cell line (SKOV3) at 0.1 nM in the presence of T cells, similar to αHer2–αCD3 using the parental mAb (Fig. 2e). However, in a low Her2-expressing cell line, lower Her2 binding variant (αHer2mA-αCD3) did not show a significant cell killing at the 0.1 nM when co-cultured with T cells while the parental and αHer2mB-αCD3 selectively demonstrated significant cell killing at the same concentration. These data support that selectivity based on target-cell antigen density can be tuned through changes in monovalent affinity and avidity, which may reduce on-target, off-tissue toxicities.

To evaluate the general applicability of this approach, we generated additional analogs using antibodies targeting other tumor-associated antigens. Specifically, we demonstrated successful application to antibodies against Cadherin-6 (CDH6) and CD33. The αCDH6–αCD3 and αCD33–αCD3 complexes were produced via disulfide bridging using the same strategy as the αHer2–αCD3 complex. The same point mutation was introduced into the Fc of each parental antibody, and each was co-expressed with αCD3–FcγRIIIa harboring the cysteine mutation.

**Fig. 3:**
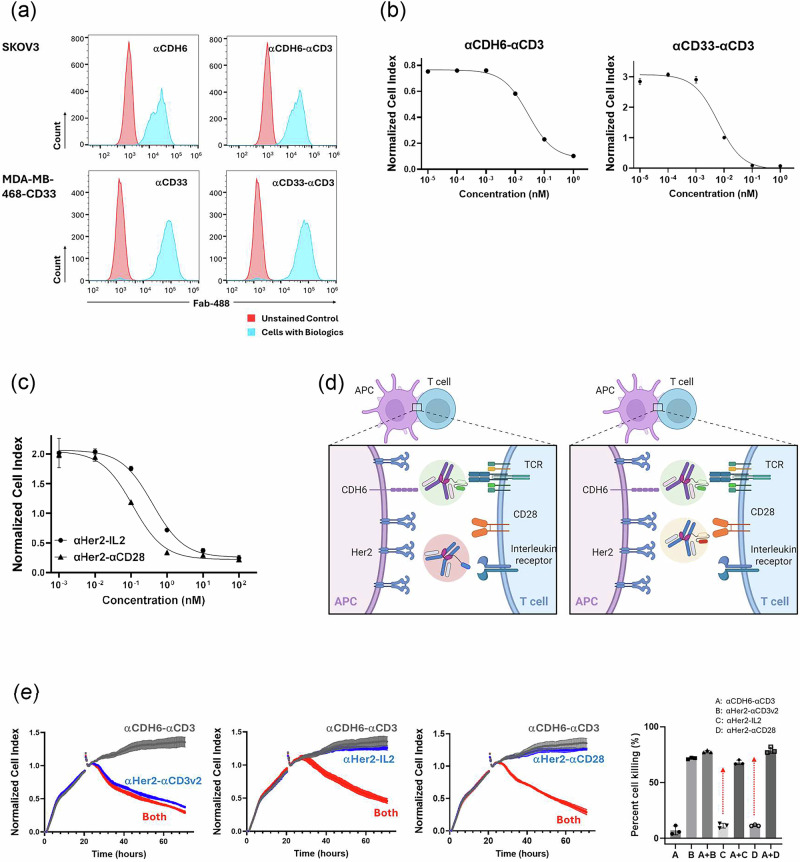
Modular application of antibody functionalization through disulfide-linked Fc-FcγR fusions. **a** Cell-binding analysis of αCDH6-αCD3 and αCD33-αCD3 complexes compared to parental antibodies, assessed by flow cytometry on SKOV3 and MDA-MB-468-CD33 cells, respectively. Fluorescent histograms are shown for non-stained cells (red) and biologic-treated cells (blue). Fab-488 indicates anti-human Fab antibody conjugated to Alexa488. **b** Normalized cell index at 48 h post-treatment plotted as a function of concentration for αCDH6-αCD3 with SKOV3 cells (left panel) and αCD33-αCD3 with MDA-MB-468-CD33 cells (right panel), in the presence of T cells at a 10:1 E:T ratio. Data are presented as mean values +/− SD from three technical replicates. **c** Normalized cell index at 48 h post-treatment plotted as a function of concentration for αHer2-IL2 (closed circle) and αHer2-αCD28 (closed triangle) complexes with SKOV3 cells, in the presence of T cells at a 10:1 E:T ratio. Data are presented as mean values +/− SD from three technical replicates. **d** Graphical illustration depicting T-cell engagement with antigen-presenting tumor cells (Her2-positive and CDH6-positive) mediated through signal 1 (TCR), signal 2 (CD28), and signal 3 (IL-2). Shown are the synergistic interactions between αCDH6-αCD3 and αHer2-IL2 (left) or αCDH6-αCD3 and αHer2-αCD28 (right). Created in BioRender. Park, M. (2026) https://BioRender.com/ytmj5y9. **e** Synergistic killing of SKOV3 cells mediated by combinations of αCDH6-αCD3 (10 pM) with either αHer2-IL2 (100 pM, middle panel) or αHer2-αCD28 (100 pM, right panel), in the presence of T cells at a 10:1 E:T ratio. For comparison, SKOV3 cell killing mediated by the combination of αCDH6-αCD3 (10 pM) and αHer2-αCD3v2 (10 pM) is shown (left panel). Percent cell killing at 48 h post-treatment is summarized for all complex combinations in the bar graph (far right). Data are presented as mean values +/− SD from three technical replicates.

The αCDH6–αCD3 complex showed comparable binding to CDH6 (Fig. 3a) and CD3 on cells (Supplementary Fig. 3a). As expected, it drove potent T cell-mediated killing of CDH6⁺ ovarian adenocarcinoma cells in vitro (Fig. 3b). Similar results were observed with the αCD33–αCD3 complex, confirming the modularity of the platform (Fig. 3a, b, Supplementary Fig. 3a).

We next explored the addition of immunomodulatory payloads to FcγRIIIa, focusing on tumor-specific “signal 2” and “signal 3” components. First, we fused an anti-CD28 scFv to FcγRIIIa and generated the αHer2–αCD28 complex. Second, we fused interleukin-2 (IL-2) to FcγRIIIa to generate the αHer2–IL2 complex. Both constructs induced concentration-dependent SKOV3 cell killing in the presence of T cells (Fig. 3c). We further confirmed that αHer2–IL2 increased pSTAT5 in CD4⁺, CD8⁺, and CD56⁺ NK cells in a dose-dependent manner (Supplementary Fig. 3b).

To evaluate whether combining distinct immune signals could enhance tumor cell killing, we tested whether co-administration of T cell engagers (signal 1) with either co-stimulatory (signal 2) or cytokine (signal 3) payloads could function in an “AND” gate configuration (Fig. 3d). SKOV3 cells were treated with αCDH6–αCD3 and αHer2–IL2 in the presence of T cells. At 10 pM αCDH6–αCD3 and 100 pM αHer2–IL2, we observed 68% cell killing—substantially higher than either agent alone (7% and 14%, respectively; Fig. 3e). A similar synergistic effect was observed with αCDH6–αCD3 and αHer2–αCD28, yielding ~80% killing compared to 7% and 13% individually. No enhancement was observed when combining αCDH6–αCD3 with αHer2–αCD3v2 (αCD3 scFv at the C-terminus of FcγRIIIa), likely due to competition for CD3 binding or receptor saturation.

**Fig. 4:**
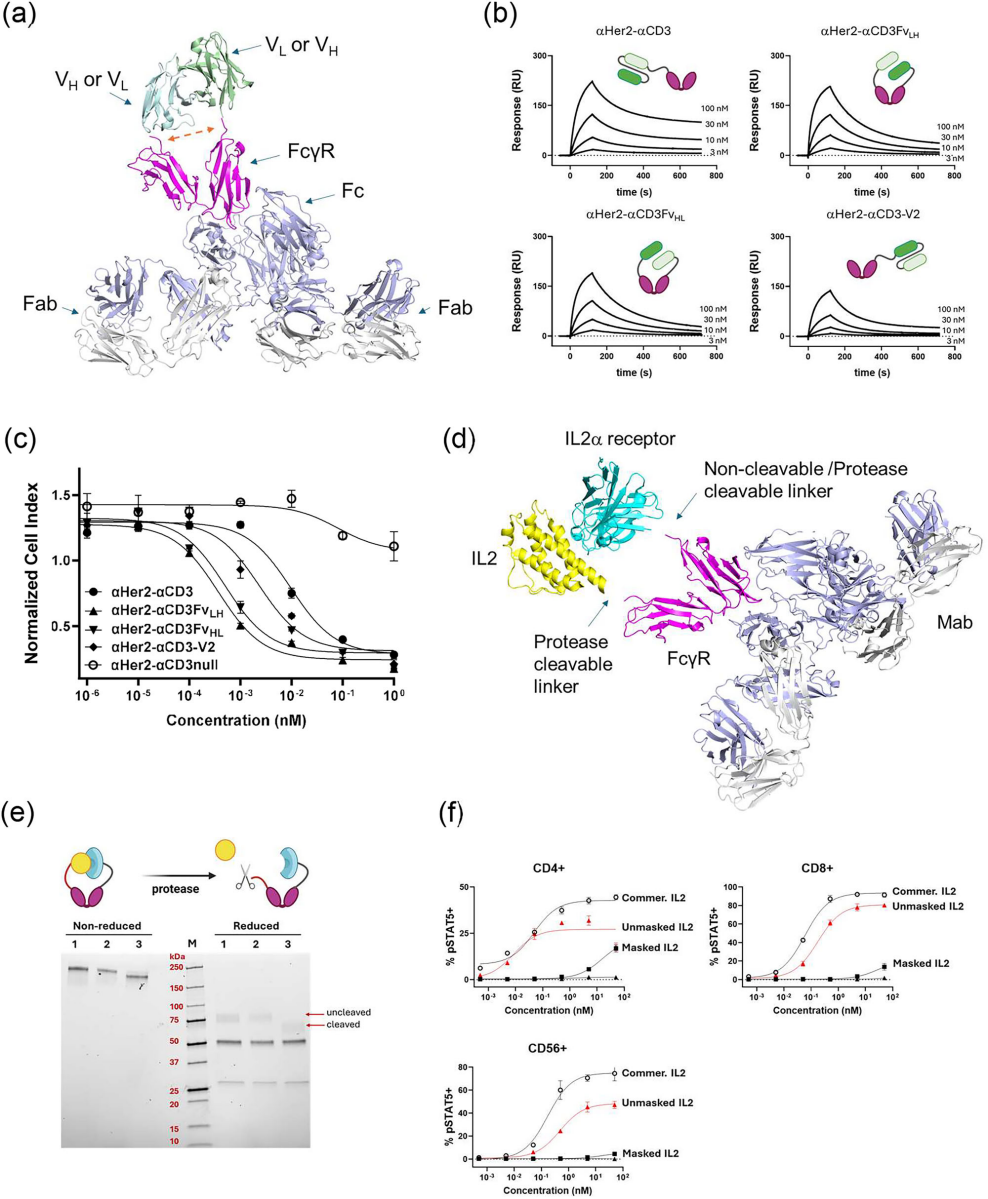
Generation of different αHer2-αCD3 complexes with various FcγR fusions and tumor-activated biologics through disulfide-linked Fc-FcγR fusions. **a** Predicted structural model of the αHer2-FcγRIIIa complex bound to αCD3 scFv, generated using AlphaFold3 (AF3). Heavy and light chains of αHer2 are depicted in light blue and light gray, respectively. FcγRIIIa is shown in magenta, and the variable heavy (VH) and variable light (VL) chains of αCD3 are shown in pale cyan and light green, respectively. The orange arrow indicates the close proximity of the N- and C-termini of FcγR. **b** Surface plasmon resonance (SPR) analysis of CD3 binding. CD3 was immobilized on an SPR chip, and varying concentrations of different αHer2-αCD3 complexes were passed over the chip surface. Response units are plotted using Biacore T200 evaluation software. Inserted figures are created in BioRender. Park, M. (2026) https://BioRender.com/f0dcj5x. **c** SKOV3 cell killing assays comparing various αHer2-αCD3 complexes with T cells (E:T ratio 10:1). Normalized cell index at 48 h post-treatment is plotted for each sample: αHer2-αCD3 (closed circle), αHer2–αCD3FvLH (closed triangle), αHer2–αCD3FvHL (closed inverted triangle), αHer2-αCD3-v2 (closed diamond), and αHer2-αCD3null (open circle). Data are presented as mean values +/− SD from three technical replicates. **d** Predicted structural model of tumor-activated αHer2-IL2 complex (αHer2-Ta-IL2) generated using AlphaFold3 (AF3). Heavy and light chains of αHer2 are shown in light blue and light gray, respectively. FcγR is depicted in magenta, with IL2 in yellow and IL2Rα in cyan. **e** SDS-PAGE analysis confirming MMP7-mediated cleavage of αHer2-Ta-IL2. Lane 1: untreated αHer2-Ta-IL2; Lane 2: αHer2-Ta-IL2 incubated overnight at 37 °C without MMP7; Lane 3: αHer2-Ta-IL2 incubated overnight at 37 °C with MMP7. The observed decrease in molecular weight after MMP7 treatment confirms successful cleavage of IL2 from the FcγR fusion. Inserted figure is created in BioRender. Park, M. (2026) https://BioRender.com/edxootz. This analysis was repeated independently three times with similar results. **f** Detection of STAT5 phosphorylation in CD8-positive and CD4-positive T cells and CD56-positive NK cells from human PBMCs (donor M46), following treatment with αHer2-Ta-IL2, αHer2-Ta-IL2 null (non-cleavable control), or αHer2-Ta-IL2 pretreated with MMP7. STAT5 phosphorylation is effectively masked in cells treated with uncleaved αHer2-Ta-IL2 (closed square) or αHer2-Ta-IL2 null (closed triangle). Unmasking IL2 via MMP7 cleavage restores STAT5 phosphorylation activity (closed red triangle). Recombinant IL2 (commercial IL2, open circle) induces robust STAT5 phosphorylation as expected. Data are presented as mean values +/− SD from three technical replicates.

To this point, we established that the intermolecular disulfide complex functions as a T cell engager when the αCD3 scFv is fused to the N-terminus of the soluble FcγR fragment. Given the importance of geometry in forming a robust immunological synapse, we explored alternative configurations. First, we fused the scFv to the C-terminus of FcγRIIIa (v2). Second, we noted from the crystal structure that the N- and C-termini of FcγRIIIa are in close proximity (Fig. 4a, orange arrow). Based on this, we asked whether we could create a “split” scFv by replacing the conventional glycine-serine linker (typically 16–20 amino acids) with FcγRIIIa I106C.

Specifically, we generated two constructs: Fv_H_–FcγRIIIa–Fv_L_ and Fv_L_–FcγRIIIa–Fv_H_. Each was co-expressed with αHer2 IgG bearing the A330C mutation and purified. Both constructs bound CD3 as determined by SPR (Fig. 4b), and each bound Jurkat cells (Supplementary Fig. 3c) as well as pan T cells from a healthy donor (M30) (Supplementary Fig. 3d). CD3 binding across all formats was comparable to the original αCD3 scFv–FcγR fusion.

Critically, we compared tumor cell killing across the panel of TCEs using SKOV3 cells and T cells (M46) at an E:T ratio of 10:1. Real-time cell analysis revealed concentration-dependent killing for all formats (Fig. 4c). Notably, the split scFv constructs were more potent. The Fv_L_–FcγRIIIa–Fv_H_ (αHer2–αCD3Fv_LH_) variant yielded the lowest EC₅₀ at 0.4 pM, while the original N-terminal fusion construct was the least potent, albeit with a still low EC_50_ of 8.1 pM (~20x less potent). These results underscore the impact of scFv orientation and configuration on biological activity.

The EC_50_ values observed across the different mAbs evaluated here were consistent with those reported for clinically approved T cell engagers (Supplementary Table 1). To directly assess in vitro cytotoxic activity relative to a clinically approved TCE, we reconstructed the BCMA-targeting T cell engager teclistamab in an Fc–FcR–based format and performed a side-by-side comparison. The Fc–FcγR–based αBCMA–αCD3 complex mediated cell killing across multiple BCMA-positive cell lines with EC_50_ values that overlapped with those of teclistamab (Supplementary Fig. 4a).

Finally, building on our prior work in tumor-activated biologics^26^ we asked whether this platform could support tumor-homing cytokine delivery. Leveraging the proximity of the N- and C-termini in FcγRIIIa, we fused IL-2 and IL-2Rα to opposite ends of FcγRIIIa to form a sterically occluded IL-2–IL-2Rα complex. We inserted a consensus matrix metalloproteinase 7 (MMP7) protease substrate between IL-2 and FcγRIIIa to enable tumor-specific activation (Fig. 4d). The resulting αHer2–Ta–IL2 construct was co-expressed, purified, and characterized.

As shown in Fig. 4e, αHer2–Ta–IL2 was cleaved by MMP7, while the non-cleavable control (αHer2–Ta–IL2–NC) remained intact (Supplementary Fig. 4b). PBMCs from a healthy donor (M46) were incubated with αHer2–Ta–IL2 ± MMP7. After overnight incubation, flow cytometry revealed pSTAT5 activation in CD4⁺, CD8⁺, and CD56⁺ cells only upon MMP7 treatment (Fig. 4f), with activity comparable to exogenous IL-2. No activation was observed with αHer2–Ta–IL2–NC ± MMP7 (Supplementary Fig. 4c), confirming protease-dependent cytokine release.

### In vivo characterization

**Fig. 5:**
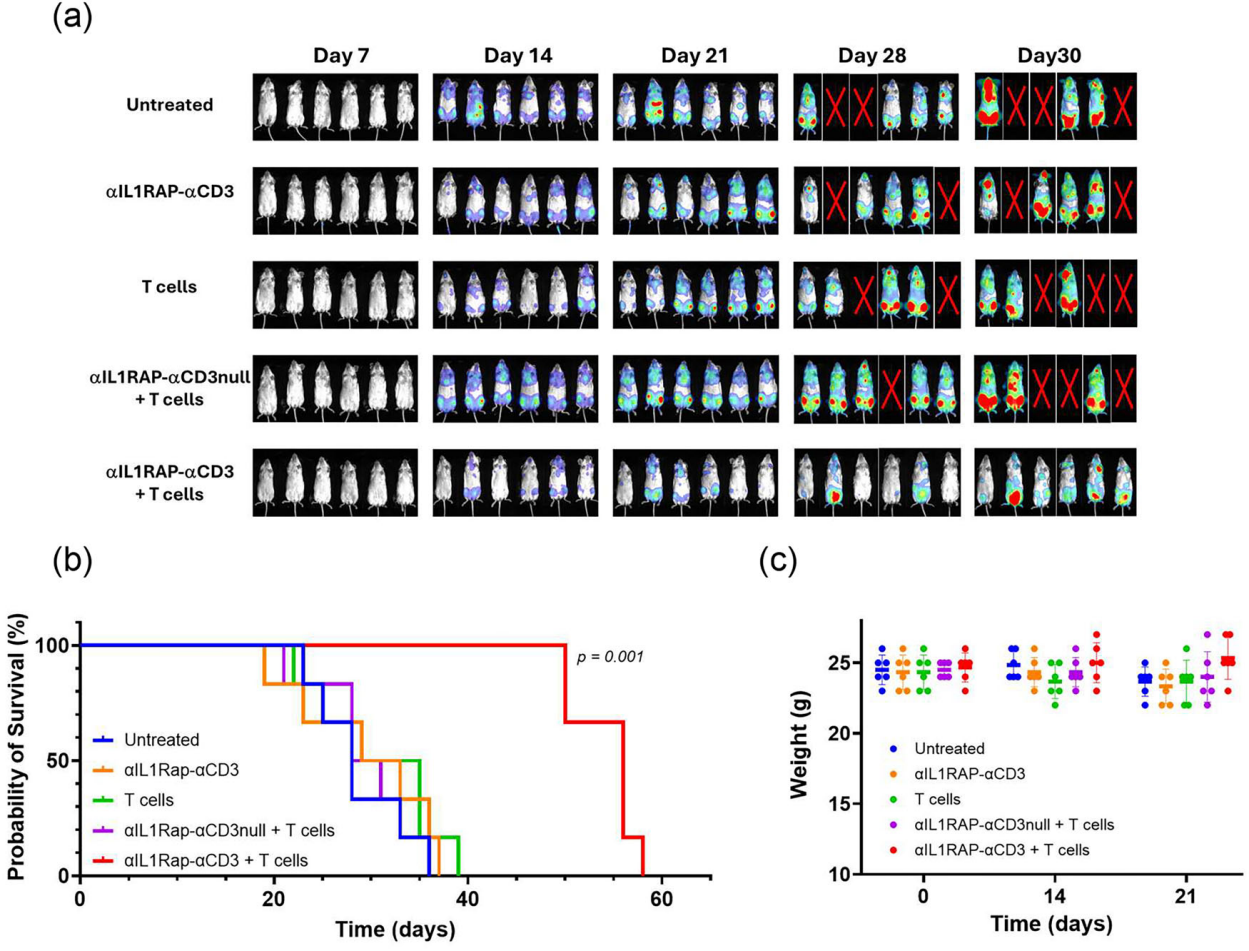
In vivo antileukemic activity of a disulfide-linked IL1RAP–T cell engager. **a** In vivo antitumor activity of αIL1RAP-αCD3 in an IL1RAP-positive Molm13GFP⁺Luc⁺ AML xenograft model in immunodeficient mice. Bioluminescence images of tumor burden for different treatment groups (untreated, αIL1RAP–αCD3 only, T cells only, αIL1RAP–αCD3 with T cells, and CD3-mutant control {αIL1RAP–αCD3null} with T cells) are shown for days 7, 14, 21, 28, and 30. Significant tumor burden reduction is observed exclusively in the group treated with αIL1RAP–αCD3 and T cells. **b** Kaplan–Meier survival curves for the different treatment groups in the Molm13GFP⁺Luc⁺ AML xenograft model. The median survival for mice treated with αIL1RAP–αCD3 and T cells is 56 days (red), while others are 28 days (untreated, blue), 31 days (αIL1RAP–αCD3 only, orange), 32 days (T cells only, green), and 29.5 days (αIL1RAP–αCD3null with T cells, violet). The *p* value of 0.001 observed for the IL1RAP–T cell engager with T cells indicates that the treatment elicited a significantly greater response than control conditions. **c** Mice body weight for the different treatment groups in the Molm13GFP⁺Luc⁺ AML xenograft model. Body weight was measured on day 0, 14 and 21. Mice treated with αIL1RAP–αCD3 and T cells (red) showed a consistent body weight, whereas the other treatment groups exhibited a modest decrease over time. Data are presented as mean values +/− SD from six mice in different groups.

To demonstrate in vivo activity of TCE through templated disulfide Fc- FcγR fusion, as proof-of-principle, we evaluated the αIL1RAP–αCD3 complex in an acute myeloid leukemia (AML) xenograft model. IL1RAP is an emerging immunotherapeutic target with high selective expression on AML blasts and leukemic stem cells (LSCs). We previously report promising results using Fab-arm exchanged IL1RAP–CD3 TCEs^27^. The αIL1RAP–αCD3 complex demonstrated target recognition on the IL1RAP positive cell line, MOLM13, and T cell driven cell killing (Supplementary Fig. 4d) in vitro. To test in vivo the antileukemic activity of the αIL1RAP–αCD3 complex generated via disulfide Fc–FcγR fusion, NSG mice were injected intravenously with 1 × 10⁶ MOLM13^GFP^⁺^Luc^⁺ cells. On day 3, 3 × 10⁶ T cells isolated from a healthy donor’s PBMCs were administered to three groups of mice: αIL1RAP–αCD3 with T cells, αIL1RAP–αCD3null (non-CD3-binding control) with T cells, and T cells alone. Two additional groups (one without any treatment and the other receiving with only αIL1RAP–αCD3) were also included in this study. αIL1RAP–αCD3 or αIL1RAP–αCD3null was administered three times per week for 42 days. Tumor burden was monitored by bioluminescence imaging. Mice treated with αIL1RAP–αCD3 and T cells showed significantly reduced tumor burden compared to control groups (Fig. 5a). The mean survival in the treated group was 56 days, compared to 28–32 days in controls (Fig. 5b).

Importantly, the αIL1RAP–αCD3null control retained IL1RAP binding but lacks CD3 engagement, and failed to reduce tumor burden or extend survival, supporting that CD3 engagement is required for therapeutic activity (Fig. 5a). The survival study was conducted as an exploratory pilot experiment to generate preliminary evidence of the therapeutic impact of αIL1RAP–αCD3 in an AML xenograft model. As the study was not designed to test a predefined hypothesis, formal power or sample size calculations were not performed. A group size of six animals per arm was selected, consistent with commonly used cohort sizes in early-stage in vivo efficacy studies, and was sufficient to enable comparative survival analysis and evaluation of treatment-associated trends. Moreover, mouse body weight was monitored over 21 days. Mice from the group treated with αIL1RAP–αCD3 with T cells showed the consistent body weight, suggesting the absence of treatment-related toxicity (Fig. 5c).

In a preliminary PK study, one of the Fc–Fcγ–based bispecific T cell engagers (αIL1RAP–αCD3) exhibited a serum half-life of 3.7 days in mice (Supplementary Fig. 4e). This extended half-life contrasts with Fc-lacking formats such as blinatumomab (approximately 2 h^28^), highlighting the advantage of Fc domain Furthermore, the observed half-life is comparable to that of FDA-approved IgG-like bispecific antibodies containing a silenced Fc region (e.g., glofitamab, 6.6 days^29^).

These proof-of-concept data demonstrate robust antitumor activity of the IL1RAP–CD3 TCE in a hematologic cancer model. Additional in vivo studies, including side-by-side comparisons with other TCE formats, are ongoing.

## Discussion

Herein, we demonstrate that the natural interaction between the Fc region of an IgG and the FcγR receptor can be leveraged to generate highly stable, multispecific antibody complexes. This approach requires no sophisticated engineering, is fully compatible with standard mammalian expression systems, and yields high levels of the desired complex (Supplementary Fig. 4f, g). The isolated complex is bivalent to the IgG target, with a single FcγR fragment featuring both N- and C-termini binding the Fc in a 2:1 manner. This configuration enables monovalent engagement of a second target (e.g., an anti-CD3 scFv), two distinct targets, or further engineering opportunities such as split scFvs or protease-activated IL-2.

Critically, valency to the target antigen is a key feature of IgGs and other immunoglobulins like IgM, as avidity plays a central role in modulating specificity, affinity, and lifetime, ultimately expanding the therapeutic window of monoclonal antibodies and multivalent biologics. Historically, FDA-approved CD3-based TCEs have been monovalent to the disease target until recently, with agents like Glofitamab (CD20-CD3 TCE with two CD20-binding Fabs) and thus have been largely limited to uniquely expressed antigens such as CD19. However, many validated disease targets (e.g., Her2, EGFR, PDL1, CD38, IL1RAP) are not exclusive to diseased cells; they are highly overexpressed in tumors but also present at lower levels in healthy tissues. This differential expression can be exploited to widen the therapeutic window. The gain in apparent affinity through avidity allows us to deliberately tune down the monomeric affinity of individual Fabs, mitigating on-target, off-tissue toxicities. Demonstrated herein, a “tuned-down” anti-Her2 biologic-CD3 TCE retained potent cytotoxicity against cells with high Her2 expression, comparable to its parental high-affinity counterpart, while showing reduced activity against cells with low Her2 levels, demonstrating improved selectivity and safety.

Notably, there are additional studies that need to be addressed prior to initiating preclinical development, including assessments of potential immunogenicity, molecular size, pharmacokinetics/pharmacodynamics (PK/PD), and the presence of an unpaired cysteine. While experimental data are still required, we anticipate that immunogenicity, at least T cell epitopes, should be minimal, as the majority of the complex is human. Beyond the linkers connecting FcγR domain and the functional domain fusions, only a single mutation in the IgG and a single mutation in FcγR are required to generate the complex. Peptide linkers, including the use of scFvs (Gly-Ser linkers) and other fusions, are commonly used in other bispecific antibodies (e.g., blinatumomab). Finally, by its very nature, the FcγR interface is blocked. Other clinical bispecific mAbs, retaining the Fc for half-life extension, require additional mutations, including in the CH3 domain of the Fc to generate heterodimers (e.g., Fab-arm exchange/knobs-into-holes), as well as LALA or STR mutations to limit endogenous FcγR interaction.

Furthermore, this approach, creating a multispecific that is bivalent to the target and monovalent to the effector cell, results in a molecule with a mass similar to that of other multispecific antibodies that are already FDA-approved (e.g., Glofitamab) or still being evaluated in clinical trials (e.g., Imvotamab, an IgM-based bispecific). Finally, given the Fc is a homodimer and a single FcγR binds to the Fc, there is a free cysteine in the “second” Fc region. This unpaired cysteine, likely capped by glutathione during production, will require subsequent modification (e.g., alkylation or acetylation) as part of the preclinical and chemistry, manufacturing, and controls (CMC) efforts needed for clinical trials to limit potential oxidation issues. Notably, unpaired cysteines have been intentionally introduced in mAbs for site-specific conjugation as part of antibody-drug conjugate strategies (e.g., ThiomAbs).

This templated disulfide strategy offers a biologically inspired and modular framework for multispecific antibody engineering. Unlike conventional bispecific formats that require extensive Fc mutations or artificial linkers, our approach leverages a native Fc–FcγR interface to achieve stable, bivalent IgG binding and monovalent effector engagement with minimal engineering. The resulting complexes retain FcRn binding, suggesting the potential for favorable pharmacokinetics, while naturally occluding FcγR interactions, eliminating the need for additional Fc silencing mutations.

Importantly, this platform is scalable and combinatorial. Any IgG can be rapidly converted into a multispecific by co-expression with engineered FcγR fusions, enabling high-throughput screening of diverse target-effector combinations. The ability to tune monovalent affinity while preserving bivalent avidity expands the therapeutic window, particularly for antigens with differential expression between tumor and healthy tissues. This strategy not only simplifies construct design and manufacturing but also opens new avenues for programmable immune engagement, payload delivery, and conditional activation, laying the groundwork for next-generation antibody therapeutics.

## Methods

### Complex production and purification

Mammalian expression vectors for Fc-IgG1 with A330C mutation and FcγRIIIa (V158 allotype) with I106C mutation were prepared separately and individually or co-transfected to ExpiCHO cells (ThermoFisher A29133) according to the manufacturer’s instructions. Mammalian expression vectors for parental antibody with A330C mutation and various FcγRIIIa fusions with I106C mutation were prepared separately and individually or co-transfected to ExpiCHO cells (ThermoFisher A29133) by manufactural instruction. Synthetic gene sequences and primers for the mutations were custom synthesized by Integrated DNA Technologies (IDT) (Supplementary Table 3). On day 7, supernatant was collected, and proteins were purified through affinity (Protein G Resin, Genscript or Ni-IMAC Resin, ThermoFisher) and size-exclusion chromatography (Cytiva) sequentially. Expected molecular weight and purity were confirmed on SDS-PAGE by the standard protocol and HPLC-SEC column (AdvanceBio SEC 300 A 2.7 μm 4.6 × 150 mm, Agilent) on 1260 Infinity II (Agilent). Mouse serum was prepared by centrifugation of the blood collected from healthy C57BL/6 mice (Charles River Laboratories) and Western blot was done by the standard protocol. SDS-PAGE and Western blot images were acquired from Biorad Image Lab (software v6.1).

### Biophysical characterization

Complex interaction with the targets were verified using SPR. Target protein was immobilized on CM5 chip (Cytiva) through Amine Coupling kit (Cytiva). Different concentrations of the complex were flown over the chip at the room temperature in HBS-EP+ buffer (Cytiva). Kinetic parameters were evaluated by Biacore T200 Evaluation Software (v3.2.1). Complex stability and folding were monitored by Differential Scanning Fluorimetry (DSF). Protein was combined with SYPRO Orange (Invitrogen) in PBS (phosphate buffered saline) in triplicates on 96-well plates (Applied Biosystems). The change in fluorescence intensity was monitored as the temperature increased from 25 to 95 °C on a QuantStudio3 Real-Time PCR (Applied Biosystems). The protein melting temperature (Tm) was determined using Protein Thermal Software v1.3 (Applied Biosystems). Human serum was acquired from Merck Millipore.

### Cell culture and T cell preparation

Human breast cancer cell lines SKOV3 (ATCC HTB-77), BT474 (ATCC HTB- 20), MDA-MB-231 (ATCC HTB-26), and MDA-MB-468-CD33^30^ were cultured in Dulbecco′s Modified Eagle Medium (DMEM) (Corning) with 10% (v/v) fetal bovine serum (FBS) (Gibco). MCF7 (ATCC HTB- 22) was cultured in Eagle’s Minimum Essential Medium (EMEM) (ThermoFisher) with 10% FBS (Gibco). Jurkat (ATCC TIB-152) and Jurkat-Lucia™ NFAT cells (InvivoGen jktl-nfat) were cultured in Iscove’s Modified Dulbecco’s Medium (IMDM) (ThermoFisher) supplemented with 10% (v/v) heat-inactivated FBS (Gibco). Human acute myeloid leukemia cell line MOLM13^GFP^⁺^Luc^⁺, ^27^ was cultured in Roswell Park Memorial Institute (RPMI) with 10% (v/v) FBS (Gemini Bio-Products), 1% penicillin/streptomycin and 2 mM glutamine. Human Myeloma cell lines MM.1S (ATCC CRL-2974), H929-GFP (gift from Dr. Pichiorri), RPMI-8226-GFPLuc^31^ were cultured in RPMI medium (Gibco) supplemented with 10% FBS (Gibco). Healthy donor peripheral blood mononuclear cells (PBMCs) were prepared using Ficoll-Paque^TM^ PLUS density gradient media (Cytiva) from the blood cones. Healthy donor blood cones were collected from participants who consented to City of Hope National Medical Center (COHNMC) and in accordance with institutional guidelines. The use of human materials in this study was reviewed and approved by the COHNMC Institutional Review Board (IRB#06229). T cells were isolated from frozen or fresh PBMCs with Pan T cell isolation kit (Miltenyi Biotec) according to the manufacturer instructions.

### In vitro characterization

Complex binding on the target cells (Jurkat, SKOV3, BT474, MDA-MB-468-CD33, MCF7, MDA-MB-231) was evaluated with flow cytometry analysis. 0.3 million cells per sample were incubated with the complex in wash buffer (PBS with 2% BSA) for 30 min at 4 °C followed by incubating with the detection antibody (Supplementary Table 2) for another 30 min at 4 °C in the dark after washing the excess amount of the complex. Cells then were washed, resuspended in wash buffer, and run on Sony SH800 flow cytometer (SONY Biotechnology). Flow cytometry analysis was performed using FSC/SSC gating to identify single cells and exclude debris, followed by SSC-A versus SSC-H gating to remove doublets. 10,000 events were collected per sample for analysis, and data was analyzed using FlowJo (version 10.10.0, BD Biosciences). Complex binding on MOLM13^GFP^⁺^Luc^⁺ was assessed analyzed on a Fortessa X-20 Cell Analyzer (BD Biosciences) with DAPI (4’,6-Diamidino-2-Phenylindole, Sigma-Aldrich) for live cell gating.

For Jurkat-NFAT-Luc activation, cancer cell lines (30,000 cells per well) were seeded in a 96 well plate (ThermoFisher). Culture media was removed after overnight incubation, 0.1 million of Jurkat-Lucia™ NFAT cells (InvivoGen) then added to each well with or without parental antibodies, FcγRIIIa fusions or complexes. The cells were further incubated for 6 h and 50 µL of luciferase substrate (InvivoGen) was applied to read the luminescent signal on Synergy 4 Microplate Reader (BioTek).

For pSTAT5 activation assay, half million PBMCs were incubated with cytokine or cytokine conjugates for 20 min at 37 °C followed by fixation over night at 4 °C. After treating cells with BD Phosflow™ Perm Buffer III (BD Biosciences) each detection Ab was individually or together added to label cells, and cells were analyzed on Attune NxT Cytometer (Invitrogen). 50,000 events were collected per sample for analysis, and data was analyzed using FlowJo (version 10.10.0, BD Biosciences). Gating strategy was shown in Supplementary Fig. 5a.

Cell adhesion and cytotoxicity for SKOV3, BT474, MDA-MB-468-CD33 and MCF7 were observed by measuring the impedance on xCELLigence RTCA MP or the impedance and imaging on xCELLigence RTCA eSight (Agilent). Adherent cells were seeded at E-plate (Agilent) at 10,000 cells per well. After 18–22 h, the cells were treated with different concentrations of the complex and human T cells at 10 to 1 an effector-to-target (E:T) ratio. T cells were isolated from human PBMCs collected from the healthy donor blood. PBMCs were prepared using Ficoll-Paque Plus (Cytiva) by the standard protocol and T cell was isolated with PanT cell Isolation kit (Miltenyi Biotec) by the manufacturer instruction. Impedance and images were monitored up to 80 h after treatment. The data was processed with RTCA Pro software of RTCA eSight Software v1.3 (Agilent).

To quantify cytokines produced through T cell engagement, target cells were seeded in 96-well plate at 10,000 cells per well. After 18–22 h, the cells were treated with different concentrations of the complex and human T cells at 10 to 1 E:T ratio. At 48-hour post-treatment, the cell culture supernatant was collected and tested on Human IFN gamma ELISA Kit (Invitrogen), Human IL-2 ELISA Kit (Acro Biosystems), and Human TNF alpha Uncoated ELISA Kit (Invitrogen) to measure IFN-γ, IL-2 and TNF-α, respectively, according to the manufacturer instruction.

For MOLM13 cell killing, cells were seeded at 30,000 cells per well in 96-well plates. Healthy donor T cells were added at E:T ratio of 3:1, along with varying concentrations of αIL1RAP–αCD3 or αIL1RAP–αCD3null complexes (0.97 to 62.5pM, 2-fold serial dilutions). At 48 h, apoptosis was evaluated with Annexin V (1:500 v/v, Invitrogen) and DAPI (0.1 µg/mL, Sigma-Aldrich) staining. Cells were washed twice with Annexin V binding buffer (BioLegend) and resuspended in the buffer containing fluorochrome-conjugated Annexin V. After 15 min of incubation at room temperature in the dark, cells were washed, resuspended in the binding buffer, and DAPI was added immediately before analysis on a Fortessa X-20 Cell Analyzer (BD Biosciences). Annexin V⁺/DAPI⁻ cells were classified as apoptotic. For MM1.S cell killing, cells are labeled with CellTrace™ Violet (CTV, ThermoFisher) and co‑cultured with isolated T cells at a 5:1 effector‑to‑target ratio, along with αBCMA-αCD3 complex or Teclistamab (TECVAYLI^®^, Johnson&Johnson) treatment on day 1. The co‑cultures are then incubated for 48 h at 37 °C with 5% CO₂. On Day 3, cells are washed and stained with Zombie NIR Live/Dead dye (BioLegend). The samples are analyzed by flow cytometry (NovoCyte Quanteon, Agilent Technologies) to measure tumor cell death. H929-GFP and RPMI-8226-GFPLuc are co-cultured with T cells without CTV labeling and tumor cells were tracked with GFP signals during analysis using FlowJo (version 10.10.0, BD Biosciences). Gating strategy was shown in Supplementary Fig. 5b.

### In vivo T cell engaged tumor eradication

MOLM13^GFP^⁺^Luc^⁺ was engrafted in 6 to 8 weeks old female NSG mice (NOD/Scid/IL-2rg⁻/⁻, The Jackson Laboratory, Stock No. 005557) through tail vein injection with 1 million cells. Thirty mice were randomly allocated to five experimental groups (*n* = 6 per group). On Day 3, 3 million T cells isolated from human PBMCs were injected intravenously (IV) followed by weekly T cell injection. 24 h after the first T cell injection, 10 µg of either αIL1RAP–αCD3 or αIL1RAP–αCD3null was injected every three days via tail vein. All the experiments and procedures were performed according to protocols approved by the Institutional Animal Care and Use Committee (IACUC) at City of Hope animal facilities (IACUC #15005). Animals were housed in specialized high-barrier, specific pathogen-free (SPF) animal rooms, with experimental and control groups co-housed. Mice were maintained on a 12-h light/12-h dark cycle at an ambient temperature of 18–23 °C and 40–60% humidity in an Association for Assessment and Accreditation of Laboratory Animal Care (AAALAC)–accredited facility. Animals were monitored daily throughout the experiment, and tumor burden was assessed by bioluminescence imaging (BLI) of luciferase-expressing MOLM13^GFP⁺Luc⁺^ cells on days 7, 14, 21, 28, and 30 post‑tumor injection. In vivo imaging was performed using the Lago X-Spectral imaging system (Spectral Instruments Imaging) and Aura Imaging software (v4.0). Mice were intraperitoneally injected with D-Luciferin (150 mg/kg; Promega) 10 min prior to imaging. Mouse survival was monitored and analyzed by Kaplan–Meier curves using the Log-rank (Mantel–Cox) test. Mice were humanely euthanized by CO₂ inhalation followed by cervical dislocation as a secondary method when predefined humane endpoints were reached. These included signs of distress such as ≥20% body weight loss, ruffled or matted fur, labored breathing, hunched posture, impaired movement or feeding, lack of response to stimuli, or neurological symptoms (e.g., twitching or tremors). Body weight was recorded on days 0, 14, and 21 for all treatment groups.

### Pharmacokinetics (PK) study in mice

6 to 8 weeks old female Ly5.1/B6 mice (Charles River Laboratories, Stock No. 564) were used to evaluate the pharmacokinetics (PK) of αIL1RAP–αCD3 in plasma. Following a single bolus dose of either 0.5 mg/kg (10 µg) or 5 mg/kg (100 µg), mice were humanely euthanized at predetermined time points (1, 4, 8, 24, 48, 72, 168, and 336 h) via CO_2_ inhalation followed by secondary euthanasia method of cervical dislocation, and plasma was collected from three animals per dose group via cardiac puncture. Forty-eight mice were used for eight time points, two doses and three mice per condition. All procedures were conducted in compliance with IACUC-approved protocols at City of Hope animal facilities (IACUC #22043). Animals were housed in pathogen-free animal holding rooms, and experimental and control groups were co-housed. Mice were maintained on 12-h light/12-h dark cycles under 18–23 °C ambient temperature with 40–60% humidity in an AAALAC–accredited animal facility. αIL1RAP–αCD3 concentrations were quantified using an enzyme-linked immunosorbent assay (ELISA). Briefly, IL1RAP (10 ng) was immobilized on a 96-well plate (Fisher Scientific) and incubated overnight at 4 °C. After washing and 2h-blocking with ELISA Blocker™ blocking buffer (ThermoFisher), plasma samples (1:5000 dilution) were added and incubated for 2 h at room temperature. The plate was then washed, followed by the addition of anti-human IgG Fc conjugated to HRP (Invitrogen) and incubation for 2 h at room temperature. After a final wash, of SuperSignal™ ELISA Pico Chemiluminescent Substrate (ThermoFisher) was added, and luminescence was measured 20 min later using CLARIOstar Plus multimode plate reader (BMG Labtech). αIL1RAP–αCD3 plasma pharmacokinetic parameters (Cmax, T1/2, and AUC) were derived from the mouse concentration-versus-time data using noncompartmental analysis methods in WinNonlin, software v8.6 (Certara).

### Model building

AlphaFold3 was used to independently generate predicted structural models for the αHer2–FcγRIIIa complex associated with the αCD3 scFv and for the tumor-activated αHer2–IL2 complex.

### Statistical analysis

GraphPad Prism software v10.4.2 was used for the statistical analysis. The unpaired t-test was used to test if the difference between two groups is statistically significant. *P* value higher than 0.05 is statistically not significant (ns). Survival differences were statistically significant (*p* < 0.05) by Log-rank (Mantel–Cox) test.

### Reporting summary

Further information on research design is available in the Nature Portfolio Reporting Summary linked to this article.

## Supplementary information

Supplementary Information

Transparent Peer Review file

Reporting Summary

Supplementary Movie 1

## Source data

Source Data

## Data Availability

The data supporting the findings of this study are available within the article and Supplementary Information file. Source data are provided with this paper.
