# Supplementary material for: Generation of mixed-valency, modular multispecific antibodies using disulfide-linked Fc–FcγR complexes
Source: Nat Commun. 2026 Apr 28;17:5821. doi: 10.1038/s41467-026-72425-5 (PMC13328583; doi:10.1038/s41467-026-72425-5)
Supplement: Supplementary file 1 — Supplementary Information [file 41467_2026_72425_MOESM1_ESM.pdf]

Supplementary information to the manuscript entitled:

Generation of mixed-valency, modular multispecific antibodies using disulfide-linked Fc–FcγR complexes

Miso Park[1], Kevin Ly[1], Bea Parcutela[1], Hyeran Choi[1], Carmina Ladra[1], Asaul Gonzalez[1], Yead Jewel[1], Hyunjun Kang[2], Melissa Valerio[2], Aparna Krishnan[3], Timothy W. Synold[3], Le Xuan Truong Nguyen[2,4], Guido Marcucci[2,4] and John C. Williams[1,*]

[1] Department of Cancer Biology and Molecular Medicine, Beckman Research Institute, City of Hope National Medical Center, Duarte, CA, USA

[2] Department of Hematologic Malignancies Translational Science, Gehr Family Center for Leukemia Research, Beckman Research Institute, City of Hope National Medical Center, Duarte, CA, USA

[3] Department of Medical Oncology and Therapeutics Research, Beckman Research Institute, City of Hope National Medical Center, Duarte, CA, USA

[4] Department of Hematology and Hematopoietic Cell Transplantation, Gehr Family Center for Leukemia Research, City of Hope National Medical Center, Duarte, CA, USA

*Corresponding Author:

John C. Williams

Department of Cancer Biology and Molecular Medicine

Beckman Research Institute, City of Hope

1500 E. Duarte Rd, Duarte, CA 91010

Tel: 626-218-1127

Email: jcwilliams@coh.org

This PDF file includes:

Supplementary Figures 1 to 5

Supplementary Tables 1 and 3

Supplementary References

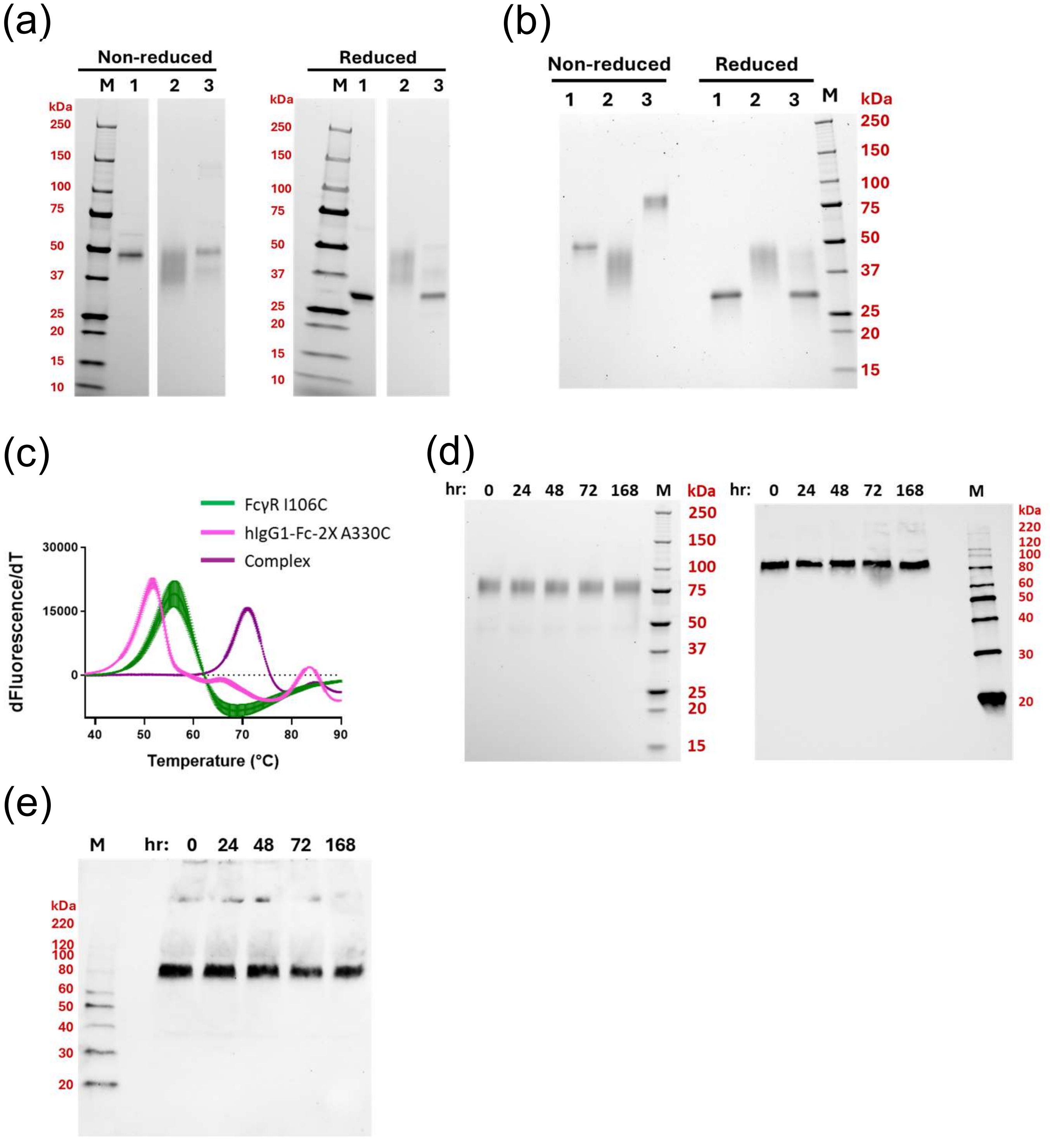

Supplementary Figure 1. Biochemical characterization and stability of Fc-FcγR fusion complexes.
(a) SDS-PAGE analysis of the Fc-FcγR complex lacking the cysteine mutation on Fc. Lane 1: Fc; Lane 2: FcγRIIIa I106C; Lane 3: Mixture of Fc and FcγRIIIa I106C; Lane M: Protein ladder.
(b) SDS-PAGE analysis of the purified Fc-FcγR complex containing affinity-enhanced mutations. Lane 1: Fc-2X A330C; Lane 2: FcγRIIIa I106C; Lane 3: Fc-2X-FcγR complex; Lane M: Protein ladder.
(c) Protein thermal shift assay. The affinity-enhanced Fc-FcγR disulfide-linked complex (purple trace) exhibits a higher melting temperature (T_M) compared to the individual protein components, indicating enhanced thermal stability. Data are presented as mean values +/- SD from three technical replicates.
(d-e) Stability assessment of Fc-FcγR complex: (d) Non-reducing SDS-PAGE of Fc-FcγR complex in PBS (left panel) and Western blot (WB) analysis (right panel) of Fc-FcγR complex stability in mouse serum over time. (e) Western blot analysis of Fc-FcγR complex stability in human serum over time. Anti-His-tag antibody-HRP was used in WB.

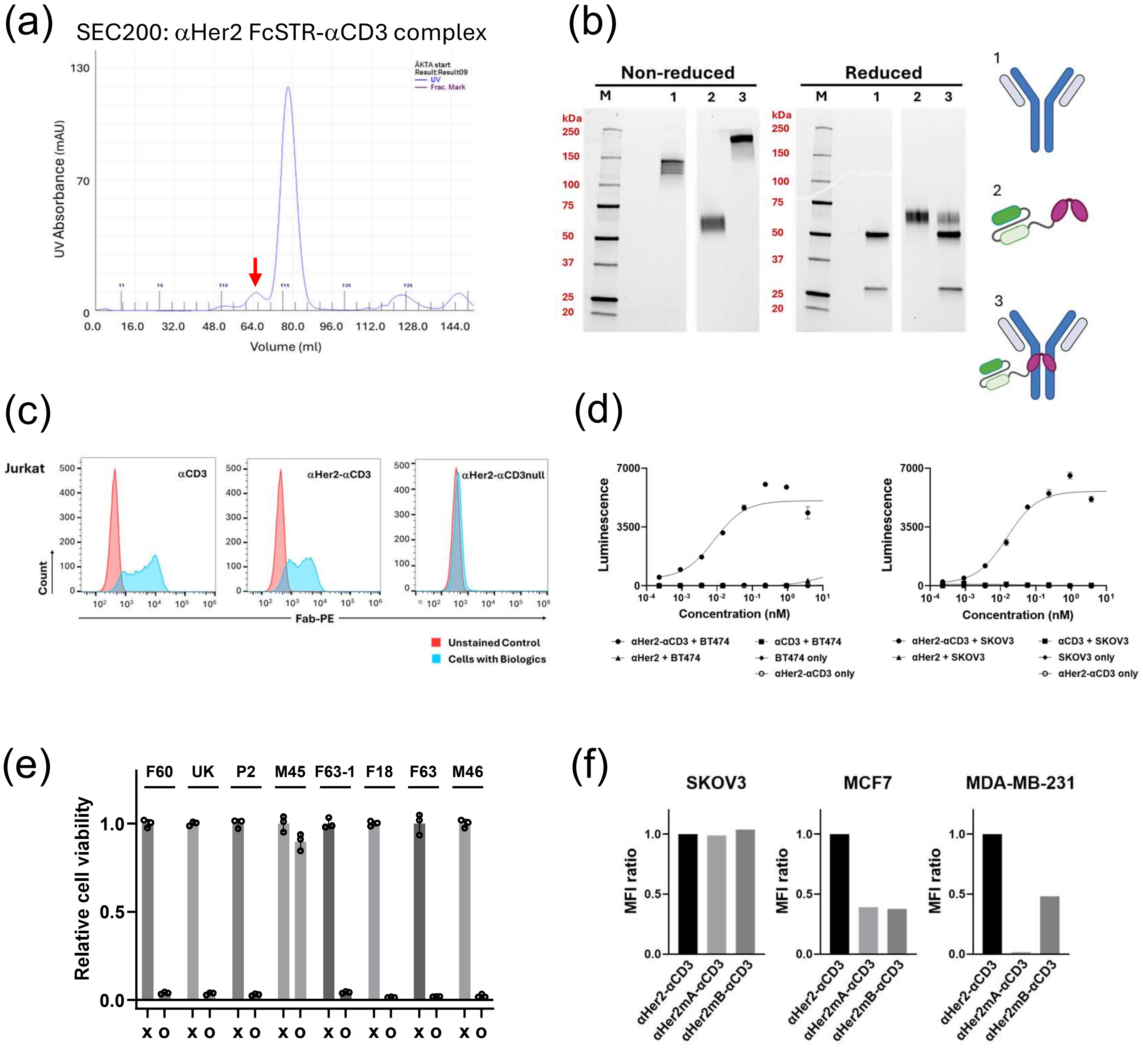

Supplementary Figure 2. Biochemical characterization and cell-based analysis of αHer2-αCD3 complexes.
(a) Size-exclusion chromatography (SEC) analysis of the αHer2 FcSTR-αCD3 complex. Co-expression of αCD3-FcγRIIIa I106C with αHer2 IgG containing A330C and additional mutations to reduce FcγRIIIa binding failed to yield a properly formed complex (indicated by the red arrow). The predominant peak observed by SEC corresponds to the αCD3-FcγRIIIa I106C fusion alone.
(b) SDS-PAGE analysis confirming the formation of the αHer2-αCD3 complex. Inserted figure is created in BioRender. Park, M. (2026) https://BioRender.com/66j3mh6
(c) Cell-binding assessment of the αHer2-αCD3 complex on Jurkat cells (CD3-positive) analyzed by flow cytometry. Unstained control cells are shown in red, and cells treated with biologics are shown in blue. Fab-PE denotes anti-human Fab antibody conjugated to PE. αHer2-αCD3null represents an αHer2 IgG disulfide complex assembled using FcγRIIIa lacking the CD3 scFv.
(d) Jurkat activation assay following co-incubation with BT474 (left panel) and SKOV3 cells (right panel). Data are presented as mean values +/- SD from three technical replicates.

(e) BT474 cell killing assays at 0.1 nM of αHer2-αCD3 with T cells isolated from eight different donor's PBMCs (x: without αHer2-αCD3 and o: with αHer2-αCD3). Relative cell viability was calculated from normalized cell index at 48 hours post-treatment against the value without αHer2-αCD3. Data are presented as mean values +/- SD from three technical replicates.
(f) Relative MFI comparison of αHer2-αCD3 complexes containing Her2-binding mutations relative to the non-mutant mAb, evaluated on SKOV3, MCF7, and MDA-MB-231 cells (N=1).

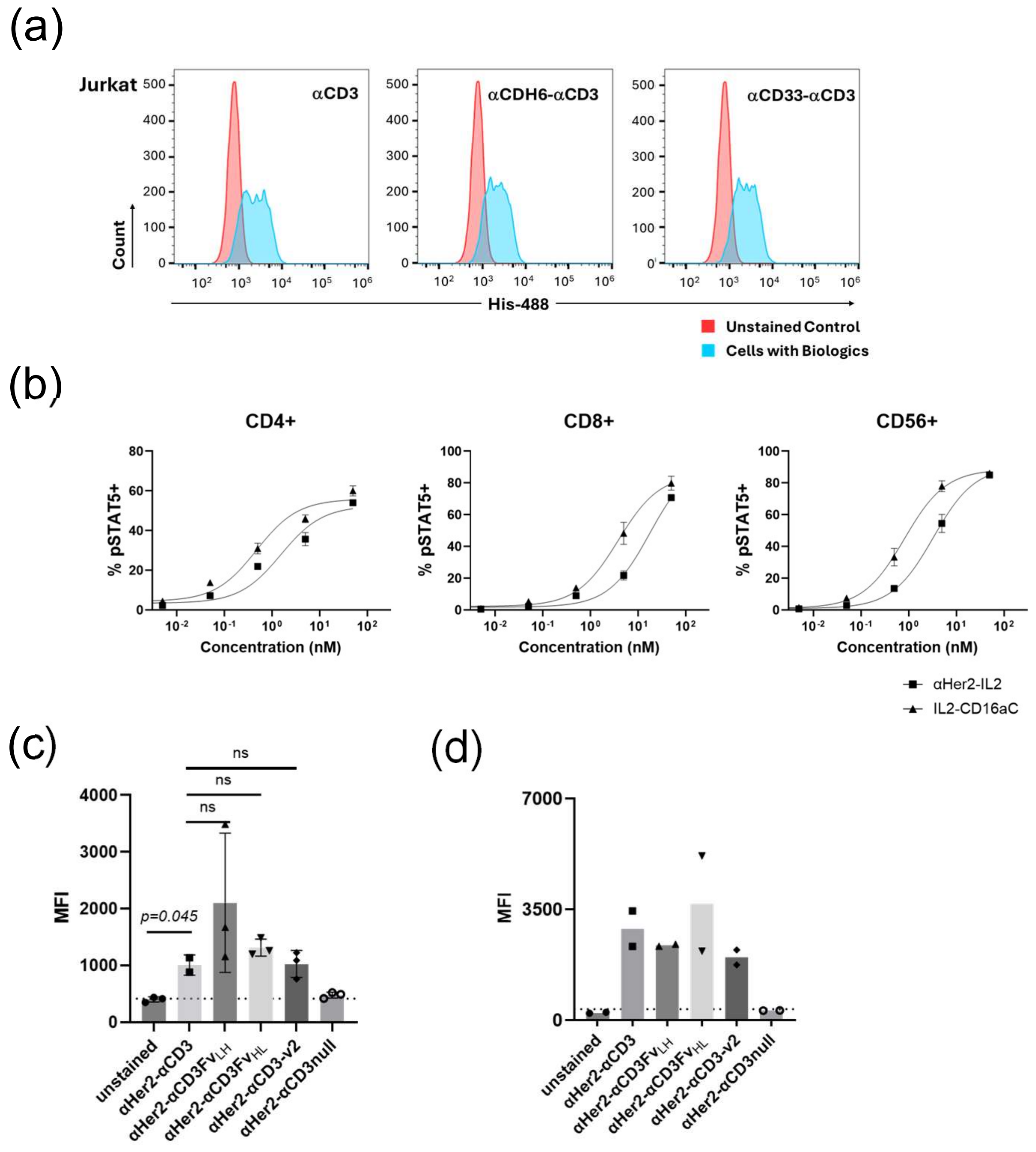

Supplementary Figure 3. Additional cell-binding analyses and functional characterization of different αHer2-αCD3 complexes.

(a) Cell-binding analysis of αCDH6-αCD3 and αCD33-αCD3 complexes to Jurkat cells. Fluorescent histograms generated by flow cytometry are shown for unstained control cells (red trace) and cells treated with each biologic (blue trace). His-488 denotes anti-His-tag antibody conjugated to Alexa488.

(b) STAT5 phosphorylation in CD4-positive and CD8-positive T cells, and CD56-positive NK cells following treatment with various concentrations of IL2-FcγRIIIa fusion (closed triangle) and αHer2-IL2 fusion (closed square). Data are presented as mean values +/- SD from three technical replicates.

(c) Mean fluorescent intensity (MFI) of various αHer2-αCD3 complexes bound to Jurkat cells (CD3-positive), detected using anti-human Fab antibody conjugated to PE (N=3). An unpaired t-test was used to assess the difference between two groups. Differences with a P value greater than 0.05 were considered not statistically significant (ns). Data are presented as mean values +/- SD from three independent replicates.

(d) MFI of different αHer2-αCD3 complexes bound to T cells isolated from human PBMCs (donor M30), detected using anti-human Fab antibody conjugated to PE. Bars represent the average MFI from two independent experiments.

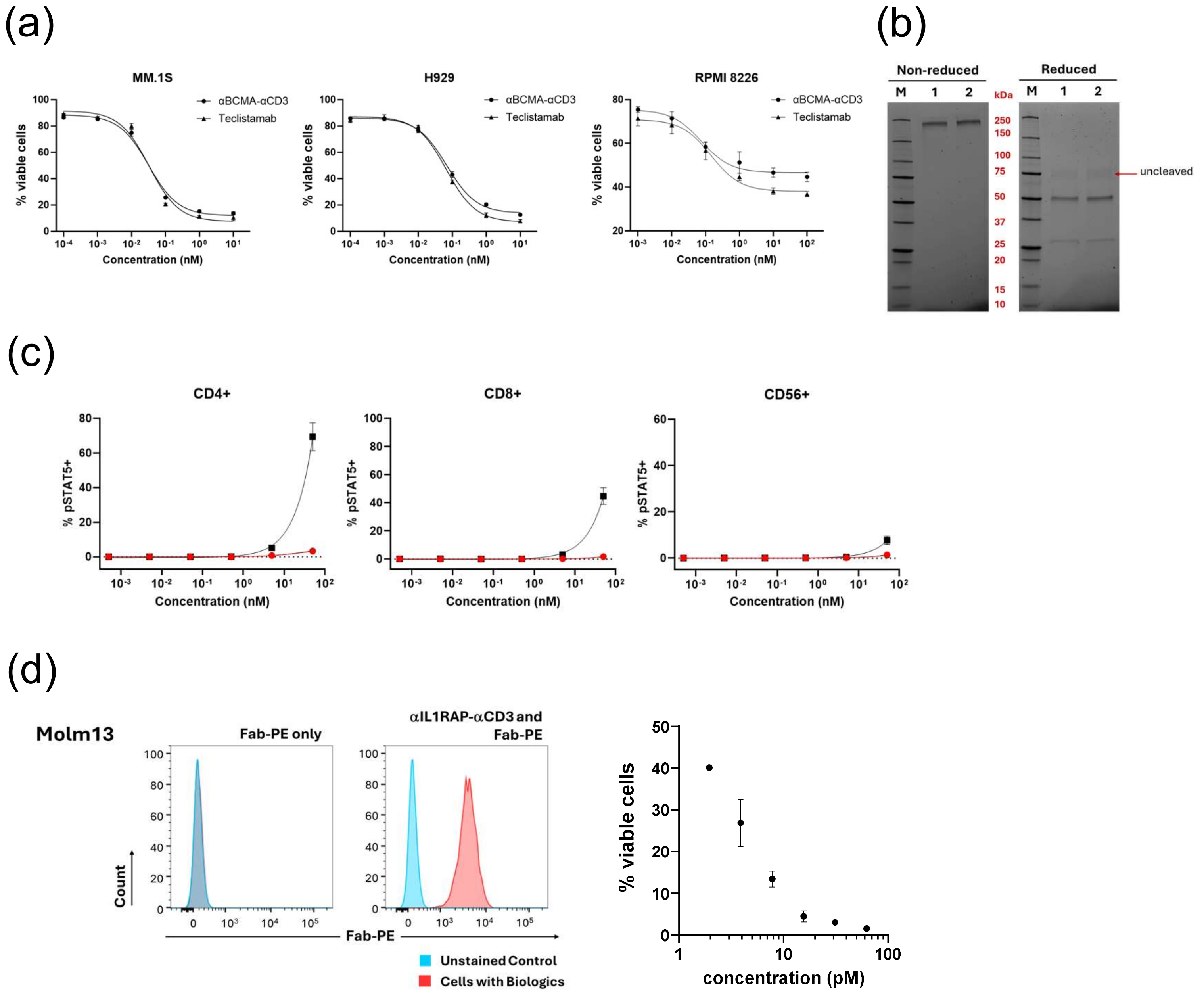

Supplementary Figure 4. Characterization and functional analysis of different IgG Fc–FcγR complexes.
(a) BCMA-positive tumor cell killing mediated by the αBCMA–αCD3 complex or the clinical BCMA-targeting bispecific T cell engager teclistamab in the presence of human T cells. Data are presented as mean values +/- SD from three technical replicates. IC50 values were calculated from three independent experiments. For MM.1S cells, IC50 values were 29 ± 13 pM for αBCMA–αCD3 and 25 ± 9 pM for teclistamab. For H929 cells, IC50 values were 103 ± 58 pM and 71 ± 9 pM, respectively. For RPMI 8226 cells, IC50 values were 56 ± 39 pM and 85 ± 67 pM, respectively.
(b) SDS-PAGE analysis of αHer2-Ta-IL2-NC following incubation without (lane 1) and with (lane 2) MMP7 cleavage.
(c) STAT5 phosphorylation (pSTAT5) detection in CD4-positive and CD8-positive T cells, as well as CD56-positive NK cells, after treatment with αHer2-Ta-IL2-NC alone (closed square), αHer2-Ta-IL2-NC incubated overnight without MMP7 (closed circle, black), and αHer2-Ta-IL2-NC incubated overnight with MMP7 (closed circle, red). Data are presented as mean values +/- SD from three technical replicates.
(d) Cell-binding and functional assessment of the αIL1RAP-αCD3 complex. Left: Cell-binding to MOLM13 cells analyzed by flow cytometry. Unstained control cells are plotted in blue, and cells treated with the biologic are plotted in red. Fab-PE denotes anti-Fab antibody conjugated to PE. Right: MOLM13 cell killing mediated by αIL1RAP-αCD3 complex in the presence of human T cells. Data are presented as mean values +/- SD from three technical replicates.

(e)

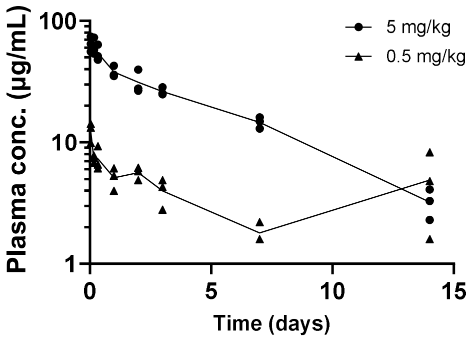

(f)

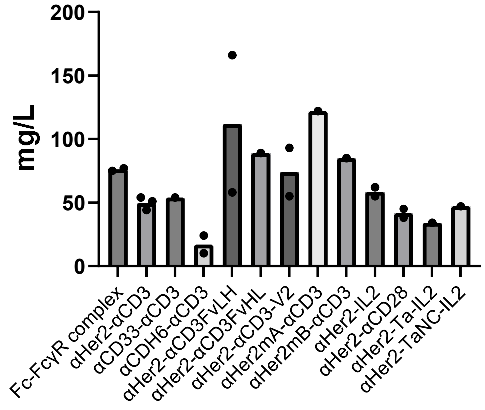

(g)

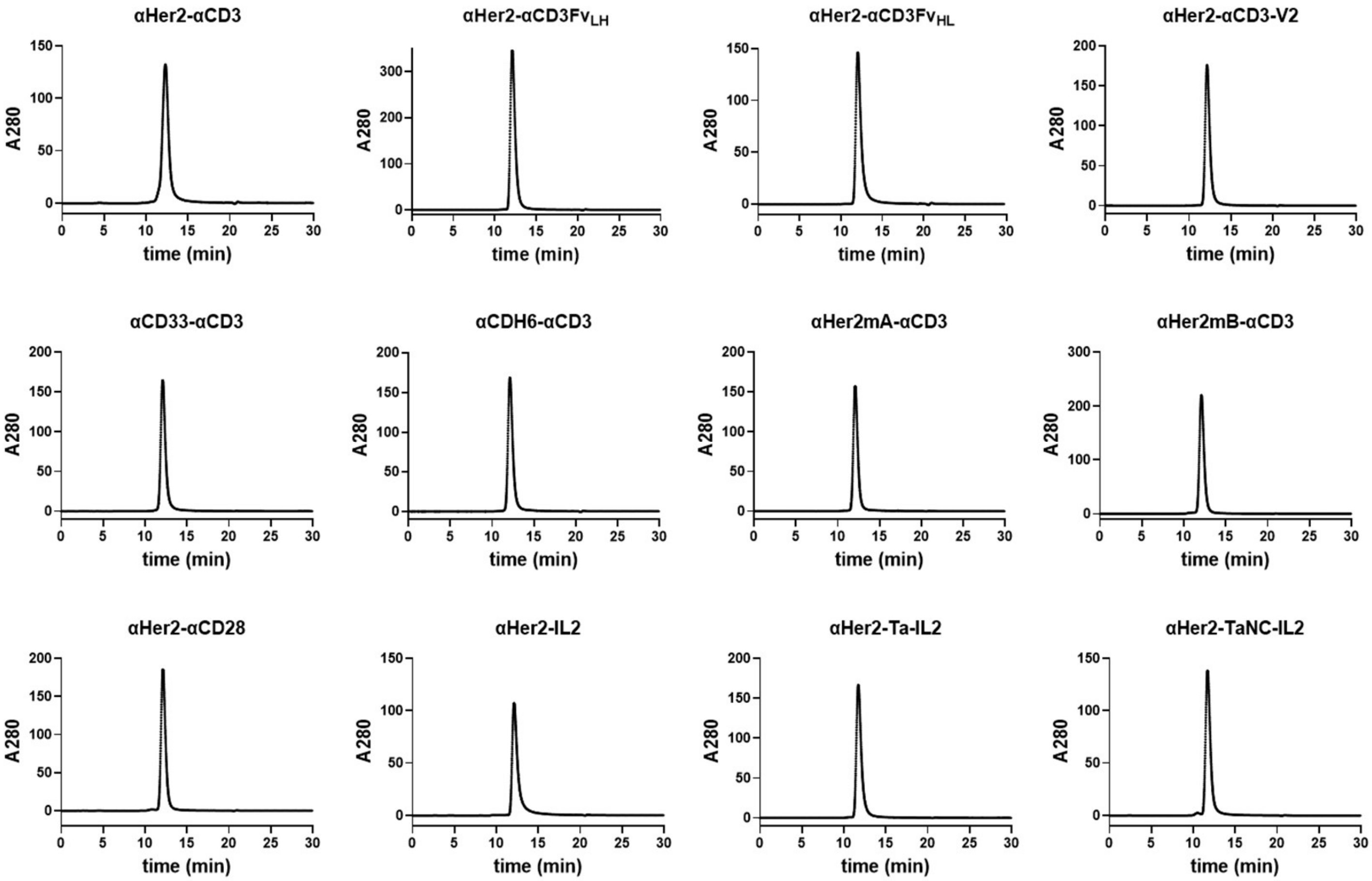

Supplementary Figure 4 conti. Characterization and functional analysis of different IgG Fc–FcγR complexes.

(e) Pharmacokinetic study of αIL1RAP-αCD3 complex. For 0.5 mg/kg dose, the average maximum serum concentration (Cmax) was 12.4 µg/mL, the area under the concentration–time curve from time zero to infinity (AUC_0–∞) was 205.8 µg/mL*day, and the terminal half-life (t½) was 3.5 days. The last time point was excluded for computing the parameters. With 5 mg/kg dose, Cmax was 65.0 µg/mL, AUC_0–∞ was 274.6 µg/mL*day, and t½ was 3.7 days. 48 mice were used for eight time points for two different doses. Data are presented as mean values from three mice per condition.

(f) Production yield of IgG Fc–FcγR complexes produced from CHO cells in this study. N=1, 2 or 3.

(g) HPLC-SEC chromatograph of IgG Fc–FcγR complexes produced from CHO cells in this study.

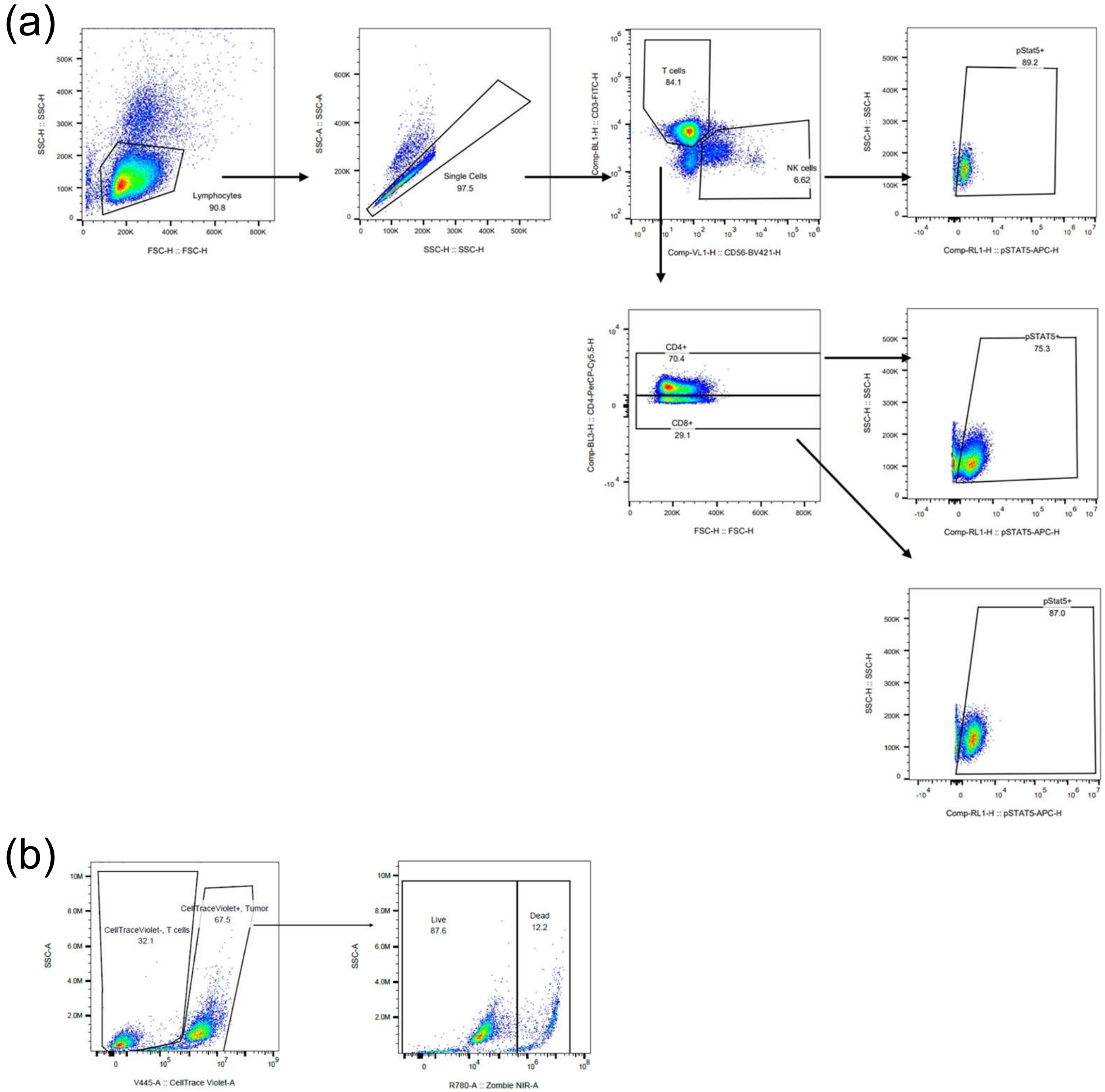

Supplementary Figure 5. Gating strategy for flow cytometry analysis.
(a) STAT5 phosphorylation in CD4 and CD8 positive T cells and NK cells from PBMCs treated with cytokine-fusion complexes, αHer2-Ta-IL2 and αHer2-Ta-IL2-NC. An initial FSC/SSC gate was applied to select the lymphocyte population and exclude cellular debris. Doublets were then removed using SSC-A versus SSC-H gating. CD4 positive T cells were selected by gating CD3+/CD56-/CD4+, and CD8 positive T cells was selected by gating CD3+/CD56-/CD4-. NK cells were selected by gating CD3-/CD56+.
(b) T cell–engaged MM.1S cell killing was assessed after 48 hours of co-culture with either the αBCMA-αCD3 complex or Teclistamab in the presence of T cells. MM.1S target cells were identified by gating on CellTrace Violet–positive (CTV^+) events, and live MM.1S cells were subsequently quantified by gating on Zombie NIR–negative ($ZombieNIR^-$) cells within the CTV^+ population.

Supplementary Tables

Supplementary Table 1. Normalized comparison of clinical T-cell engagers

Molecule	Format	Target(s)	Reported EC_{50}	Normalized (pM)	Ref.
Blinatumomab	scFv BiTE	CD19×CD3	20–414 pg/mL	0.2–4.1 pM	1
AMG330	scFv BiTE	CD33×CD3	0.4–3 pM	0.4–3 pM	2
AMG701	HLE BiTE	BCMA×CD3	~19 pM	~19 pM	3
Epcoritamab	IgG1 DuoBody	CD20×CD3	0.073–2.40 pM	0.073–2.40 pM	4
Glofitamab	2:1 IgG	CD20×CD3	low-pM	1–10 pM est.	5
Mosunetuzumab	IgG	CD20×CD3	—	sub-pM to low-pM	6
Teclistamab	IgG4	BCMA×CD3	0.06–0.45 nM	60–450 pM	7
Talquetamab	IgG4	GPRC5D×CD3	0.206–0.612 nM	206–612 pM	8

- All EC_{50} values normalized to picomolar (pM)

- BiTE MW ~50 kDa; IgG MW ~150 kDa

- Values remain assay-dependent; normalization enables format-level comparison

Supplementary Table 2. List of detection antibodies used in this study

Name	Manufacturer	Catalog number	Clone number	Dilution used	Lot number	RRID
Mouse anti-Human IgG Fab Secondary Antibody, PE	Invitrogen	MA1-10377	4A11	1:100	AC4653 582	AB_2536766
Alexa Fluor® 488 anti-His Tag Antibody	BioLegend	652509	J099B12	1:1000	B439714	AB_2716151
c-Myc Monoclonal Antibody (9E10)	Invitrogen	MA1-980	9E10	1:100	AB4090 41	AB_558470
Goat anti-Mouse IgG (H+L) Highly Cross-Adsorbed Secondary Antibody, Alexa Fluor™ Plus 488	Invitrogen	A32723	Polyclon al	1:500	XC3433 55	AB_2866489
6x-His Tag Monoclonal Antibody (HIS.H8), HRP	Invitrogen	MA1-21315-HRP	HIS.H8	1:1000	3239537	AB_2536989
Alexa Fluor® 647 Mouse Anti-Stat5 (pY694)	BD Biosciences	612599	pY694	1:10	0279919	AB_399882
Brilliant Violet 421™ anti-human CD56 (NCAM) Antibody	BioLegend	362552	5.1H11	1:10	B432214	AB_2566061
PerCP/Cyanine5.5 anti-human CD4 Antibody	BioLegend	357414	A161A1	1:10	B391216	AB_2565666
FITC anti-human CD3 Antibody	BioLegend	300440	UCHT1	1:10	B279209	AB_2562046
Goat anti-Human IgG F(ab')2 Secondary Antibody, HRP	Invitrogen	31482	HP6017	1:1000	UC2821 10	AB_429694

Supplementary Table 3. Fc-IgG1 and FcγRIIIa sequences and primers for the mutations

	Sequence
Fc-IgG1	GACAAAACTCACACATGCCCACCGTGCCCAGCACCTGAACTCCTGGGG GGACCGTCAGTCTTCCTCTTCCCCCCAAAACCCAAGGACACCCTCATGA TCTCCCGGACCCCTGAGGTCACATGCGTGGTGGTGGACGTGAGCCACGA AGACCCTGAGGTCAAGTTCAACTGGTACGTGGACGGCGTGGAGGTGCA TAATGCCAAGACAAAGCCGCGGGAGGAGCAGTACAACAGCACGTACCG GGTGGTCAGCGTCCTCACCGTCCTGCACCAGGACTGGCTGAATGGCAAG GAGTACAAGTGCAAGGTCAGCAACAAAGCCCTCCCAGCCCCCATCGAG AAAACCATCTCCAAAGCCAAAGGGCAGCCCCGAGAACCACAGGTGTAC ACCCTGCCCCCATCCCGGGATGAGCTGACCAAGAACCAGGTCAGCCTG ACCTGCCTGGTCAAAGGCTTCTATCCCAGCGACATCGCCGTGGAGTGGG AGAGCAATGGGCAGCCGGAGAACAACTACAAGACCACGCCTCCCGTGC TGGACTCCGACGGCTCCTTCTTCCTCTACAGCAAGCTCACCGTGGACAA GAGCAGGTGGCAGCAGGGGAACGTCTTCTCATGCTCCGTGATGCATGA GGCTCTGCACAACCACTACACGCAGAAGTCCCTCTCCCTGTCTCCGGGT AAATAG
FcγRIIIa (V158 allotype)	AGGACCGAGGACTTGCCCAAGGCAGTTGTTTTTCTGGAGCCCCAGTGGT ACAGAGTTCTTGAAAAAGATTCAGTGACCTTGAAGTGCCAAGGGGCTTA CTCACCGGAAGACAACTCTACTCAGTGGTTCCACAACGAGTCCTTGATT TCATCTCAGGCCTCTAGTTATTTCATAGATGCTGCAACAGTCGATGACTC TGGCGAGTACAGATGTCAAACTAACTTGTCCACCCTCTCCGATCCAGTA CAATTGGAGGTCCACATTGGTTGGCTCCTCCTCCAAGCGCCCAGATGGG TGTTTAAGGAAGAAGACCCCATTCATCTGCGCTGTCATTCCTGGAAAAA TACGGCACTTCACAAAGTCACATATCTGCAAAACGGGAAAGGCCGCAA GTATTTCCACCATAATTCCGACTTTTATATTCCCAAAGCCACTCTCAAAG ACAGCGGCTCCTACTTCTGCCGGGGGCTCGTCGGGTCAAAGAACGTAAG CTCCGAAACCGTGAACATCACTATTACCCAAGGC
Fc-A330C-1	AGCCCTCCCATGCCCCATCGAGAAAAC
Fc-A330C-2	TTGTTGCTGACCTTGCACT
CD16a-I106C-1	GGAGGTCCACTGTGGTTGGCTCC
CD16a-I106C-2	AATTGTACTGGATCGGAG

Supplementary References

1. U.S. Food and Drug Administration. Blinatumomab (Blincyto): BLA #125557. https://www.accessdata.fda.gov/drugsatfda_docs/nda/2014/125557Orig1s000PharmR.pdf (2014).
2. Friedrich, M. et al. Preclinical Characterization of AMG 330, a CD3/CD33-Bispecific T-Cell–Engaging Antibody with Potential for Treatment of Acute Myelogenous Leukemia. *Mol Cancer Ther*. **13,** (2014)
3. Goldstein, R.L. et al. AMG 701 induces cytotoxicity of multiple myeloma cells and depletes plasma cells in cynomolgus monkeys. *Blood Adv.* **4,** 4180-4194 (2020).
4. U.S. Food and Drug Administration. Epcoritamab (Epkinly): BLA #761324. https://www.accessdata.fda.gov/drugsatfda_docs/nda/2023/761324Orig1s000MultidisciplineR.pdf (2023).
5. U.S. Food and Drug Administration. Glofitamab-gxbm (Columvi): BLA #761309. https://www.accessdata.fda.gov/drugsatfda_docs/nda/2023/761309Orig1s000MultidisciplineR.pdf (2023).
6. U.S. Food and Drug Administration. Mosunetuzumab (Lunsumio): BLA #761263. https://www.accessdata.fda.gov/drugsatfda_docs/nda/2023/761263Orig1s000MultidisciplineR.pdf (2023).
7. Pillarisetti, K. et al. Teclistamab is an active T cell–redirecting bispecific antibody against B-cell maturation antigen for multiple myeloma. *Blood Adv.* **4,** 18 (2020).
8. U.S. Food and Drug Administration. Talquetamab-tgvs (Talvey): BLA # 761342. https://www.accessdata.fda.gov/drugsatfda_docs/nda/2023/761342Orig1s000MultidisciplineR.pdf (2023).
