# Supplementary material for: Generation of mixed-valency, modular multispecific antibodies using disulfide-linked Fc–FcγR complexes
Source: Nat Commun. 2026 Apr 28;17:5821. doi: 10.1038/s41467-026-72425-5 (PMC13328583; doi:10.1038/s41467-026-72425-5)
Supplement: Supplementary file 3 — Reporting Summary [file 41467_2026_72425_MOESM3_ESM.pdf]

nature portfolio

Corresponding author(s): John C. Williams

Last updated by author(s): Mar 18, 2026

Reporting Summary

Nature Portfolio wishes to improve the reproducibility of the work that we publish. This form provides structure for consistency and transparency in reporting. For further information on Nature Portfolio policies, see our Editorial Policies and the Editorial Policy Checklist.

Statistics

For all statistical analyses, confirm that the following items are present in the figure legend, table legend, main text, or Methods section.

n/a	Confirmed	
☐	☒	The exact sample size (*n*) for each experimental group/condition, given as a discrete number and unit of measurement
☐	☒	A statement on whether measurements were taken from distinct samples or whether the same sample was measured repeatedly
☐	☒	The statistical test(s) used AND whether they are one- or two-sided<br>*Only common tests should be described solely by name; describe more complex techniques in the Methods section.*
☒	☐	A description of all covariates tested
☒	☐	A description of any assumptions or corrections, such as tests of normality and adjustment for multiple comparisons
☐	☒	A full description of the statistical parameters including central tendency (e.g. means) or other basic estimates (e.g. regression coefficient) AND variation (e.g. standard deviation) or associated estimates of uncertainty (e.g. confidence intervals)
☐	☒	For null hypothesis testing, the test statistic (e.g. *F*, *t*, *r*) with confidence intervals, effect sizes, degrees of freedom and *P* value noted<br>*Give P values as exact values whenever suitable.*
☒	☐	For Bayesian analysis, information on the choice of priors and Markov chain Monte Carlo settings
☒	☐	For hierarchical and complex designs, identification of the appropriate level for tests and full reporting of outcomes
☒	☐	Estimates of effect sizes (e.g. Cohen's *d*, Pearson's *r*), indicating how they were calculated

Our web collection on statistics for biologists contains articles on many of the points above.

Software and code

Policy information about availability of computer code

Data collection	Biacore T200 Control Software (v3.2.1), QuantStudio Design & Analysis Software (v1.5.1), Agilent OpenLab CDS ChemStation software (vC.01.10), Sony Cell Sorter Software (v2.1.5), Attune NxT Software (v6.2.3), Agilent RTCA eSight (v1.3), BD FACSDiva Software (v9.0), Agilent NovoExpress (v1.6.2), Aura Imaging software (v4.0)
Data analysis	Biacore T200 Evaluation Software (v3.2.1), Biorad Image Lab software (v6.1), Applied Biosystems Protein Thermal Shift Software (v1.3), GraphPad Prism Software (v10.4.0 and v10.4.2), Agilent OpenLab CDS ChemStation software (vC.01.10), FlowJo (v10.10.0), Agilent RTCA eSight (v1.3), WinNonlin software (v8.6), AlphaFold3, PyMOL (v2.5.5)

For manuscripts utilizing custom algorithms or software that are central to the research but not yet described in published literature, software must be made available to editors and reviewers. We strongly encourage code deposition in a community repository (e.g. GitHub). See the Nature Portfolio guidelines for submitting code & software for further information.

Data

Policy information about availability of data

All manuscripts must include a data availability statement. This statement should provide the following information, where applicable:

- Accession codes, unique identifiers, or web links for publicly available datasets
- A description of any restrictions on data availability
- For clinical datasets or third party data, please ensure that the statement adheres to our policy

The data supporting the findings of the study are available within the article and Supplementary Information file and from the corresponding author upon

reasonable request.

Research involving human participants, their data, or biological material

Policy information about studies with human participants or human data. See also policy information about sex, gender (identity/presentation), and sexual orientation and race, ethnicity and racism.

Reporting on sex and gender	Sex or gender has been reported for healthy donor PBMCs and in vivo study. Sex or gender was not considered in study design and no determination was ovserved based on sex and gender.
Reporting on race, ethnicity, or other socially relevant groupings	This information has not been collected.
Population characteristics	This information has not been collected.
Recruitment	This information has not been collected.
Ethics oversight	This information has not been collected.

Note that full information on the approval of the study protocol must also be provided in the manuscript.

Field-specific reporting

Please select the one below that is the best fit for your research. If you are not sure, read the appropriate sections before making your selection.

☒ Life sciences ☐ Behavioural & social sciences ☐ Ecological, evolutionary & environmental sciences

For a reference copy of the document with all sections, see nature.com/documents/nr-reporting-summary-flat.pdf

Life sciences study design

All studies must disclose on these points even when the disclosure is negative.

Sample size	In vitro cell experiment carried out with technical duplicated or triplicates, and one to three biological replicates were collected and analyzed. For evaluating T cell engaged cell killing, seven additional different donor T cells were used. For in vivo tumor eradication, thirty mice were used in five experimental groups, with n = 6 per group. For PK study, 48 mice were used in sixteen groups with n=3 per group.
Data exclusions	The last time point from the group with 0.5 mg/kg dose was excluded for computing the parameters in PK study.
Replication	Biological replicates were collected and measured at different dates and times.
Randomization	For in vivo tumor eradication, thirty mice were randomly allocated to five experimental groups, with n = 6 per group. For PK study, 48 mice were randomly divided into sixteen groups, with n=3 per group.
Blinding	Blinding was not feasible in this in vivo study due to the distinct injection regimens required for each treatment group.

Reporting for specific materials, systems and methods

We require information from authors about some types of materials, experimental systems and methods used in many studies. Here, indicate whether each material, system or method listed is relevant to your study. If you are not sure if a list item applies to your research, read the appropriate section before selecting a response.

Materials & experimental systems

n/a	Involved in the study	
☐	☒	Antibodies
☐	☒	Eukaryotic cell lines
☒	☐	Palaeontology and archaeology
☐	☒	Animals and other organisms
☒	☐	Clinical data
☒	☐	Dual use research of concern
☒	☐	Plants

Methods

n/a	Involved in the study	
☒	☐	ChIP-seq
☐	☒	Flow cytometry
☒	☐	MRI-based neuroimaging

Antibodies

Antibodies used	Mouse anti-Human IgG Fab Secondary Antibody, PE (4A11) (Invitrogen, MA1-10377, lot AC4653582, 1:100 dilution), Alexa Fluor® 488 anti-His Tag Antibody (J099B12) (BioLegend, 652509, lot B439714, 1:1,000 dilution), c-Myc Monoclonal Antibody (9E10) (Invitrogen,

MA1-980, lot AB409041, 1:100 dilution), Goat anti-Mouse IgG (H+L) Highly Cross-Adsorbed Secondary Antibody, Alexa Fluor™ Plus 488 (Invitrogen, A32723, lot XC343355, 1:500 dilution), 6x-His Tag Monoclonal Antibody (HIS.H8), HRP (Invitrogen, MA1-21315-HRP, lot 3239537, 1:1,000 dilution), Alexa Fluor® 647 Mouse Anti-Stat5 (pY694) (BD Biosciences, 612599, lot 0279919, 1:10 dilution), Brilliant Violet 421™ anti-human CD56 Antibody (5.1H11) (BioLegend, 362552, lot B432214, 1:10 dilution), PerCP/Cyanine5.5 anti-human CD4 Antibody (A161A1) (BioLegend, 357414, lot B391216, 1:10 dilution), FITC anti-human CD3 Antibody (UCHT1) (BioLegend, 300440, lot B279209, 1:10 dilution), Mouse anti-Human IgG Fc Secondary Antibody, HRP (HP6017) (Invitrogen, 05-4220, lot UC282110, 1:1,000 dilution)

Validation

Each primary or secondary antibodies used in this study were validated on manufacturers quality certifications.

Eukaryotic cell lines

Policy information about cell lines and Sex and Gender in Research

Cell line source(s)

SKOV3 (ATCC HTB-77), BT474 (ATCC HTB- 20), MDA-MB-231 (ATCC HTB-26), MCF7 (ATCC HTB- 22), Jurkat (ATCC TIB-152), and MM.1S (ATCC CRL-2974) were acquired from ATCC. Jurkat-Lucia™ NFAT cells were obtained from InvivoGen. MDA-MB-468-CD33 was from the published study (Kuo, Y.C. et al. Antibody-based redirection of universal Fabrack-CAR T cells selectively kill antigen bearing tumor cells. J. Immunother. Cancer 10, {2022}.). MOLM13GFP⁺Luc⁺ was from the published study (Zhang, Y. et al. IL1RAP-specific T cell engager depletes acute myeloid leukemia stem cells. J. Hematol. Oncol. 17, 67 {2024}). H929-GFP is a gift from Dr. Pichiorri. RPMI-8226-GFPLuc was from the published study (Viola, D. et al. Daratumumab induces mechanisms of immune activation through CD38+ NK cell targeting. Leukemia 16, 35 {2020}). T cells were isolated from healthy donor peripheral blood mononuclear cells (PBMCs) from healthy donor blood cones collected at City of Hope National Medical Center (COHNMC) under IRB#06229. ExpiCHO cell was purchased from ThermoFisher (A29127).

Authentication

None of the cell lines used were authenticated.

Mycoplasma contamination

Cell lines were not tested for mycoplasma contamination.

Commonly misidentified lines
(See ICLAC register)

None

Animals and other research organisms

Policy information about studies involving animals; ARRIVE guidelines recommended for reporting animal research, and Sex and Gender in Research

Laboratory animals

6 to 8 weeks old female NSG mice (NOD/Scid/IL-2rg^{-}/$^{-}$, Stock No. 005557) were obtained from the Jackson Laboratory. 6 to 8 weeks old female Ly5.1/B6 mice (Stock No. 564) were obtained from Charles River Laboratories.

Wild animals

The study did not involve wild animals.

Reporting on sex

Female mice were used in the study.

Field-collected samples

The study did not involve field-collected samples.

Ethics oversight

All the experiments and procedures were performed according to protocols approved by the Institutional Animal Care and Use Committee (IACUC) at City of Hope animal facilities (IACUC #15005 and IACUC #22043).

Note that full information on the approval of the study protocol must also be provided in the manuscript.

Plants

Seed stocks

The study does not involve the use of plant material.

Novel plant genotypes

Describe the methods by which all novel plant genotypes were produced. This includes those generated by transgenic approaches, gene editing, chemical/radiation-based mutagenesis and hybridization. For transgenic lines, describe the transformation method, the number of independent lines analyzed and the generation upon which experiments were performed. For gene-edited lines, describe the editor used, the endogenous sequence targeted for editing, the targeting guide RNA sequence (if applicable) and how the editor was applied.

Authentication

Describe any authentication procedures for each seed stock used or novel genotype generated. Describe any experiments used to assess the effect of a mutation and, where applicable, how potential secondary effects (e.g. second site T-DNA insertions, mosiacism, off-target gene editing) were examined.

Flow Cytometry

Plots

Confirm that:

- [x] The axis labels state the marker and fluorochrome used (e.g. CD4-FITC).
- [x] The axis scales are clearly visible. Include numbers along axes only for bottom left plot of group (a 'group' is an analysis of identical markers).
- [ ] All plots are contour plots with outliers or pseudocolor plots.
- [x] A numerical value for number of cells or percentage (with statistics) is provided.

Methodology

Sample preparation	For complex binding on target cells,.0.3 million cells per sample were incubated with the complex in wash buffer (PBS with 2% BSA) for 30 minutes at 4 °C followed by incubating with the proper detection antibodies for another 30 minutes at 4°C in the dark. Washing was done in between. For pSTAT5 activation assay, 0.5 million PBMCs were incubated with complex for 20 min at 37 °C followed by fixation over night at 4 °C. Cells were treated with BD Phosflow™ Perm Buffer III (BD Biosciences) and incubated with each detection antibodies. For Molm13 killing, 30,000 cells were treated with the complex/T cells for 48hr, washed twice with Annexin V binding buffer, and incubated for 15min at room temperature in the dark with fluorochrome-conjugated Annexin V. After washing and resuspension in binding buffer, DAPI was added immediately before analysis. For MM1.S killing, cells are labeled with CellTrace™ Violet (CTV, ThermoFisher) first and treated with the complex/T cells for 48hr. On Day 3, cells are washed and stained with Zombie NIR Live/Dead dye (BioLegend) and analyzed by flow cytometry. H929-GFP and RPMI-8226-GFPLuc are co-cultured with T cells without CTV labeling and tumor cells were tracked with GFP signals and Zombie NIR Live/Dead dye (BioLegend).
Instrument	SH800S (Sony Biotechnology) for cell binding, Attune NxT Cytometer (Invitrogen) for pSTAT5 activation assay, Fortessa X-20 Cell Analyzer for MOLM13 cell killing, and NovoCyte Quanteon (Agilent Technologies) for MM1.S/H929/RPMI cell killing.
Software	Sony Cell Sorter Software (v2.1.5), Attune NxT Software (v6.2.3), BD FACSDiva Software (v9.0), Agilent NovoExpress (v1.6.2), FlowJo (v10.10.0)
Cell population abundance	These cells were not sorted.
Gating strategy	The gated population is indicated in the Supplementary Figure Legend and Methods.

- [x] Tick this box to confirm that a figure exemplifying the gating strategy is provided in the Supplementary Information.
