# Supplementary material for: Generation of mixed-valency, modular multispecific antibodies using disulfide-linked Fc–FcγR complexes
Source: Nat Commun. 2026 Apr 28;17:5821. doi: 10.1038/s41467-026-72425-5 (PMC13328583; doi:10.1038/s41467-026-72425-5)
Supplement: Supplementary file 4 — Supplementary Movie 1 [file 41467_2026_72425_MOESM4_ESM.pptx]

## Slide 1
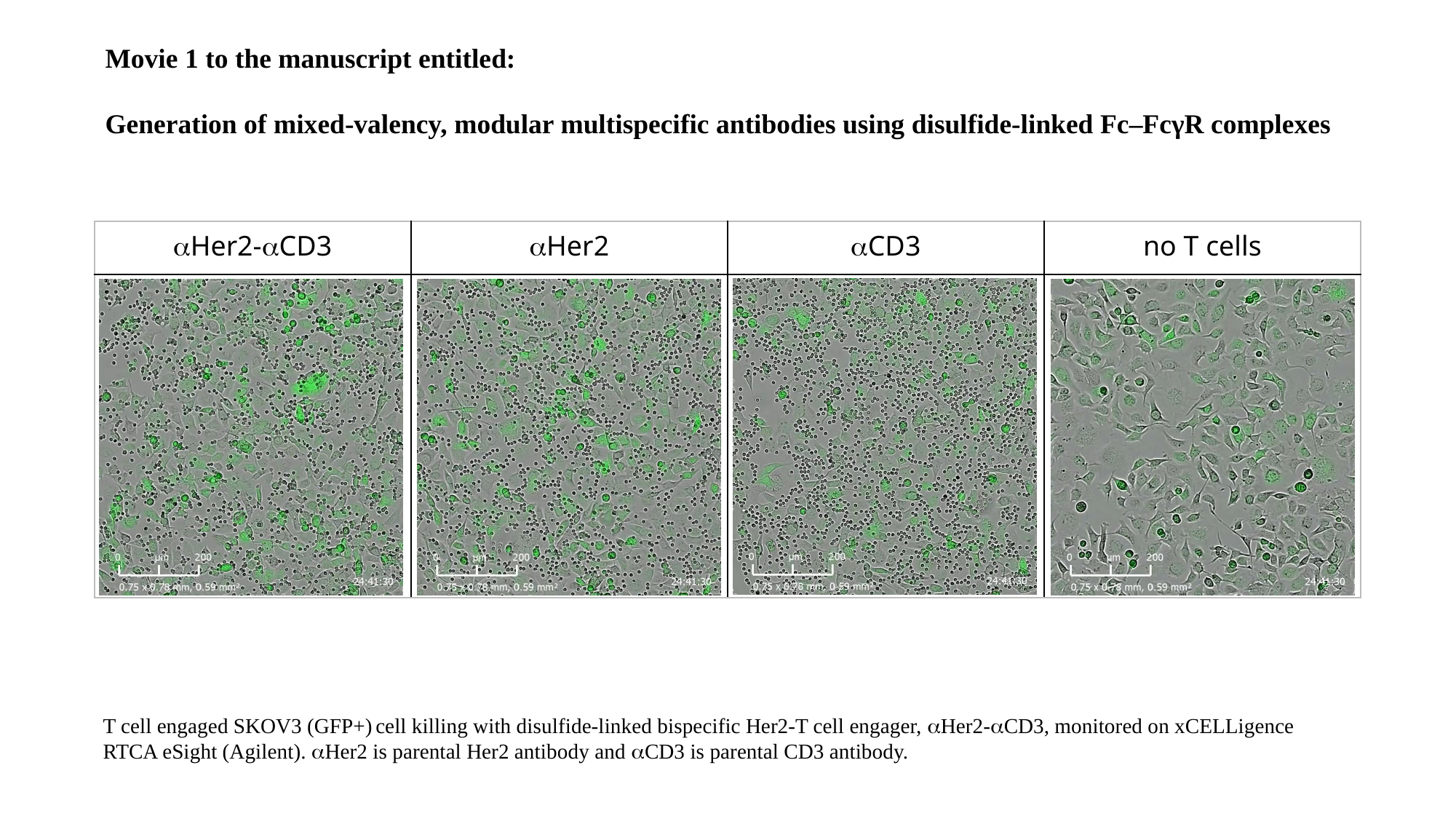

Movie 1 to the manuscript entitled:
Generation of mixed-valency, modular multispecific antibodies using disulfide-linked Fc–FcγR complexes
| αHer2-αCD3 | αHer2 | αCD3 | no T cells |
| --- | --- | --- | --- |
| | | | |
T cell engaged SKOV3 (GFP+) cell killing with disulfide-linked bispecific Her2-T cell engager, αHer2-αCD3, monitored on xCELLigence RTCA eSight (Agilent). αHer2 is parental Her2 antibody and αCD3 is parental CD3 antibody.
