# Supplementary material for: Generation of mixed-valency, modular multispecific antibodies using disulfide-linked Fc–FcγR complexes
Source: Nat Commun. 2026 Apr 28;17:5821. doi: 10.1038/s41467-026-72425-5 (PMC13328583; doi:10.1038/s41467-026-72425-5)
Supplement: Supplementary file 2 — Transparent Peer Review file [file 41467_2026_72425_MOESM2_ESM.pdf]

nature portfolio

Peer Review File

Generation of mixed-valency, modular multispecific antibodies using disulfide-linked Fc–FcγR complexes

Corresponding Author: Professor John Williams

This file contains all reviewer reports in order by version, followed by all author rebuttals in order by version.

Version 0:

Reviewer comments:

Reviewer #1

(Remarks to the Author)
In this manuscript, the authors present the in vitro and in vivo activity of a covalent fusion of the extracellular domain of Fc receptor gamma III and Fc, which are connected with a single disulphide bond, designed on the natively interacting binding partners, for whom the interaction is however only micromolar. The authors realized the opportunity of creating bispecific reagents by fusing single-chain antibodies to the CD16 ECD, while keeping the original specificity of the antibody. As both N-and C-termini of the CD16 ECD are free, the format offers potential for diverse fusions with variable valency.
Formation of the constructs was monitored with SDS-PAGE and their stability with DSF, accelerated stability assay and incubation in serum. Interestingly, the complexes were formed when a high-affinity CD16-binding Fc was used, but not when with the silenced Fc variant.
Then trastuzumab was used as the “Fc-partner”, and an anti-CD3 single chain was fused to the N-terminus of CD16 ECD. The affinities to the original antigens were as retained, and the molecule could interact with the neonatal Fc receptor, promising an extended half-life in vivo. The cosnstruct could mediate specific activation of the NFAT-luciferase expressing Jurkat cells. Using donor cells, Her2-positive tumor cell killing was induced, and activation of T cells was shown by monitoring their cytokine profile.
Affinity-reduced trastuzumab variants were also tested and it was shown that by affinity modulation, only the recognition of strongly-antigen positive cells can be achieved.
To show the general applicability of the platform, two other antibodies were used as “Fc-partners”, and interaction with the relevant cell type as well as T-cell mediated killing was achieved for both anti-Cadherin 6 and anti-CD33 antibody candidate.
Further, the authors explored more options of functionalization of the construct by adding (fusing) the costimulatory agonistic anti-CD28 or IL-2. Both constructs induced concentration-dependent Her-2 positive cell killing in the presence of T cells. The effect of combining the signals was studied by testing the combinations of anti-CDH6–anti-CD3 and anti-Her2–IL2, as well as antiCDH6–antiCD3 with antiHer2–antiCD28, and more potent T-cell mediated killing was observed.
A further interesting construct was created by fusing the VL and VH domains separately to each terminus of CD16 ECD. The split single chain constructs were more potent in the killing assays than those with a conventional GS-rich linker.
Then the authors were interested to probe their scaffold molecule as a carrier of cytokine activity that could be conditionally activated. They fused IL-2 and IL-2Rα to opposite ends of FcγRIIIa to form a sterically occluded complex, and incorporated MMP-7 recognition sites. pSTAT5 activation as a measure of IL-2 stimulation was measured in $CD4^+$, $CD8^+$, and $CD56^+$ cells only upon MMP7 treatment, indicating selective cytokine release.
In vivo activity was also demonstrated. In this case, the antibody was directed against IL1Rap. T-cell activity against MOLM13 cells was monitored with tumor burden measurement with bioluminescence imaging. The variant of the antibody with a „silenced“ anti-CD3, which retains IL1Rap binding but lacks CD3 engagement, was used as a control. Bispecific antibodies indeed reduced tumor burden as well as extended the survival, and their activity was dependent on the CD3 engagement.
This is a very elegant study with well designed and well presented experiments. The reasoning is straightforward and easy to follow. Because of the high potential translational value of the novel construct, I would ask the authors to comment on the following points:
- Yields of the molecule expected from a standard mammalian expression system.
- The Figures with description of biophysical properties of the fusion proteins would be improved if the HPLC-SEC analysis in native conditions is shown (best for all described constructs).
- In vivo experiment: Monitoring of body weight of experimental animals to show the potential toxic effects of test molecules

would be good additional information here (Figure 5).
- Can it be expected that the disulphide bond in the fusion protein is affected by high concentrations of HSA (with the single Cys34) in serum, such as seen in cysteine-coupled ADCs? There are higher molecular weight complexes forming after 3 days serum incubation, was their identity discovered?

Please find below a list of remarks which I hope will be helpful.

Line 30: „complex forms a 2:1 stoichiometry" – in the case of homodimeric Fc such statements can always be misunderstood, although I realize that this is the usual description – it could be mentioned once that this is one homodimeric Fc with one ligand molecule.
Lines 43-33: "functionalize" or "imbue them with novel functionality"- would you consider rewording?
Line 92: Numbering scheme for immunoglobulins and the PDB ID should be mentioned at this point.
Line 123: Leu 330 –should be Ala or is something wrong with the numbering
Figure 1: HPLC-SEC in native conditions would be a good addition to this Figure.
Line 260: "substantially higher than either agent alone" – how many experiments were performed, could the statistical significance be shown?
Supplementary Figure 3 cd. In 3c, the experiment has only N=1, and in 3d it is not so clear if the differences between groups could be significant if the deviations were not so large – please could you supplement the data so that these experiments are on the level with the rest of the manuscript.
Line 443:" the detection antibody" – please specify the reagent, including its RRID identifier

Reviewer #2

(Remarks to the Author)
In this manuscript the authors seek to repurpose the native IgG Fc–FcγR interaction into a universal, covalent docking site to generate multispecific antibodies.
They show that introducing the disulfide between A330C on the Fc and I106C on FcγRIIIa elicits stable, covalent complexes which support addition of payloads such as anti-CD3, anti-CD28, IL-2, and protease-activated IL-2. They then explore the activity of these molecules in vitro and in vivo.
This is a novel and innovative approach which appears to deliver a flexible plug and play technology. The experiments are in the main clear and generate conclusive data. The attraction is the flexibility of the approach and simple modifications that permit this.

However, some areas of the manuscript require clarification and/or improvement.

The introduction states that "we recognized that the FcgR extracellular regions specifically bind to the Fc region of an IgG1 naturally and with relatively high affinity (~1 nM to 1 uM) seemingly unwilling to document that FcgR1 is a high affinity receptor for IgG1 and the others significantly lower.
Why was FcgR1 not selected given the stated aim?

Given the manuscript reports a new technology with claims of overcoming issues of production etc – statements such as "Both proteins were expressed at high levels and remained stable throughout purification." require substantiation with data.

To assess the stability of the complex under physiologically relevant conditions, the authors incubated the purified disulfide-linked complex in PBS and in mouse serum at 37 °C for 7 days. An additional control would be to perform these studies with human serum (the construct is based on human FcgRs and human IgG and so human serum is more relevant).

The SPR data showing retained binding to FcRn is informative but should be supported with in vitro cell binding studies and/or in vivo assessment of persistence. The authors clearly identify areas for further work (immunogenicity, PK/PD). I agree much of this is outside the remit of this initial paper but think the PK is a fundamental point that should be addressed as it would limit utility of the approach.

Some of the terminology used is a little misleading – e.g. "First, the Her2 disulfide complex was generated using FcγRIIIa without the CD3 scFv fusion (αHer2–αCD3mut)." This implies a mutated CD3 scFv not one that is absent – perhaps CD3-null or similar would be more appropriate?

In general there is a lack of benchmarking versus other TCEs for the in vitro and in vivo activity studies.

Figure 1C: there are some extremely broad bands in the SDS PAGE. What are they the result of? This should be commented upon

Figure 2 and elsewhere cell experiments are performed with "n=2 donors with triplicates generated " – this is ambiguous as to what is being shown in the figures, with no additional insight in the legend. I presume it is a technical triplicate from a single donor. For robustness and estimation of real biological variation at least n=3 should be shown with the error bars reflecting the SD from the means of the triplicate values.

As commented in the discussion, valency is a key feature of IgG and most approved TCES are monovalent. However, 2+1 and other modalities have been developed as the authors describe with Glofitamab. The authors demonstrate nicely with

their Her2 biologic the potential of their approach for rapidly generating multivalent molecules.

Minor:
Some typos/errors exist: e.g. on line 91: “identify residues at the interact that”

Version 2:

Reviewer comments:

Reviewer #1

(Remarks to the Author)
In the revised version of the manuscript, the authors have added data on biophysical characterization of the novel constructs, on their pharmacokinetics by determining half-life in mouse studies in vivo, and on the direct toxicity in vivo studies by including data on weight monitoring of experimental animals. Multiple measurements of cell binding data as determined by FACS are also shown for a higher significance of results, and detail level of the Methods section was improved for higher reproducibility. All novel data support author's arguments.
Minor typos:
Line 127: “We initially focused on Leu330 in the Fc“ – this should be Ala330
Line 353: „(approximately 2 hours28)“ – reference 28 not in the correct format
Line 356: reference currently listed as 9 at this point should very probably be 29 (describing PK of glofitamab)

Reviewer #2

(Remarks to the Author)
The authors have done an excellent job in addressing my initial comments and have significantly improved the manuscript. i have only 1 remaining issue: the rebuttal letter describes that "We emphasize that work to extend the platform to FcγRI is ongoing, and our early data suggest that this will be feasible with additional engineering. We have added clarifying text to the manuscript" - i coudldnt find any text relating to this

Open Access This Peer Review File is licensed under a Creative Commons Attribution 4.0 International License, which permits use, sharing, adaptation, distribution and reproduction in any medium or format, as long as you give appropriate credit to the original author(s) and the source, provide a link to the Creative Commons license, and indicate if changes were made.
In cases where reviewers are anonymous, credit should be given to 'Anonymous Referee' and the source.
The images or other third party material in this Peer Review File are included in the article’s Creative Commons license, unless indicated otherwise in a credit line to the material. If material is not included in the article’s Creative Commons license and your intended use is not permitted by statutory regulation or exceeds the permitted use, you will need to obtain permission directly from the copyright holder.

To view a copy of this license, visit https://creativecommons.org/licenses/by/4.0/

RESPONSE TO REVIEWERS' COMMENTS

Reviewer #1 (Remarks to the Author):
In this manuscript, the authors present the in vitro and in vivo activity of a covalent fusion of the extracellular domain of Fc receptor gamma III and Fc, which are connected with a single disulphide bond, designed on the natively interacting binding partners, for whom the interaction is however only micromolar. The authors realized the opportunity of creating bispecific reagents by fusing single-chain antibodies to the CD16 ECD, while keeping the original specificity of the antibody. As both N-and C-termini of the CD16 ECD are free, the format offers potential for diverse fusions with variable valency. Formation of the constructs was monitored with SDS-PAGE and their stability with DSF, accelerated stability assay and incubation in serum. Interestingly, the complexes were formed when a high-affinity CD16-binding Fc was used, but not when with the silenced Fc variant.

Then trastuzumab was used as the “Fc-partner”, and an anti-CD3 single chain was fused to the N-terminus of CD16 ECD. The affinities to the original antigens were as retained, and the molecule could interact with the neonatal Fc receptor, promising an extended half-life in vivo. The cosnstruct could mediate specific activation of the NFAT-luciferase expressing Jurkat cells. Using donor cells, Her2-positive tumor cell killing was induced, and activation of T cells was shown by monitoring their cytokine profile.

Affinity-reduced trastuzumab variants were also tested and it was shown that by affinity modulation, only the recognition of strongly-antigen positive cells can be achieved.

To show the general applicability of the platform, two other antibodies were used as “Fc-partners”, and interaction with the relevant cell type as well as T-cell mediated killing was achieved for both anti-Cadherin 6 and anti-CD33 antibody candidate.

Further, the authors explored more options of functionalization of the construct by adding (fusing) the costimulatory agonistic anti-CD28 or IL-2. Both constructs induced concentration-dependent Her-2 positive cell killing in the presence of T cells. The effect of combining the signals was studied by testing the combinations of anti-CDH6–anti-CD3 and anti-Her2–IL2, as well as antiCDH6–antiCD3 with antiHer2–antiCD28, and more potent T-cell mediated killing was observed.

A further interesting construct was created by fusing the VL and VH domains separately to each terminus of CD16 ECD. The split single chain constructs were more potent in the killing assays than those with a conventional GS-rich linker.

Then the authors were interested to probe their scaffold molecule as a carrier of cytokine activity that could be conditionally activated. They fused IL-2 and IL-2Rα to opposite ends of FcγRIIIa to form a sterically occluded complex, and incorporated MMP-7 recognition sites. pSTAT5 activation as a measure

of IL-2 stimulation was measured in $CD4^+$, $CD8^+$, and $CD56^+$ cells only upon MMP7 treatment, indicating selective cytokine release.

In vivo activity was also demonstrated. In this case, the antibody was directed against IL1Rap. T-cell activity against MOLM13 cells was monitored with tumor burden measurement with bioluminescence imaging. The variant of the antibody with a „silenced" anti-CD3, which retains IL1Rap binding but lacks CD3 engagement, was used as a control. Bispecific antibodies indeed reduced tumor burden as well as extended the survival, and their activity was dependent on the CD3 engagement.

This is a very elegant study with well designed and well presented experiments. The reasoning is straightforward and easy to follow. Because of the high potential translational value of the novel construct, I would ask the authors to comment on the following points:

Thank you for your thoughtful review and valuable insights. Your comments have greatly improved the quality of our manuscript. We have incorporated all suggested revisions into the updated version. Below, we address each point in detail, with our responses highlighted in blue for clarity.

- Yields of the molecule expected from a standard mammalian expression system.

Production yields for all constructs are now provided in Supplementary Fig. 4d. IgG Fc–FcγR complexes expressed, transiently, in CHO cells produced between ~28 and ~40 mg/L depending on the construct, with the αHer2–FcγR complex yielding ~40 mg/L. The αHer2–Ta–IL2 fusion, which contains an additional cytokine domain, expressed at ~12 mg/L. These data have been added to the main manuscript.

- The Figures with description of biophysical properties of the fusion proteins would be improved if the HPLC-SEC analysis in native conditions is shown (best for all described constructs).

HPLC-SEC chromatograms of all described constructs are included in Figure 1 (Fig. 1d) and Supplementary Figures (Supplementary Fig. 4e), and the main manuscript has been updated accordingly.

- In vivo experiment: Monitoring of body weight of experimental animals to show the potential toxic effects of test molecules would be good additional information here (Figure 5).

Body weight measurements for all treatment groups is now included in Figure 5 (Fig.5c), and the main manuscript has been updated accordingly.

- Can it be expected that the disulphide bond in the fusion protein is affected by high concentrations of HSA (with the single Cys34) in serum, such as seen in cysteine-coupled ADCs? There are higher molecular weight complexes forming after 3 days serum incubation. Was their identity discovered?

The disulfide bond in our Fc–FcγR complex is unlikely to undergo exchange with HSA Cys34. In cysteine-coupled ADCs, disulfide reshuffling occurs because the conjugated drug is not naturally associated with the antibody and the engineered cysteine is solvent-exposed. In contrast, in our system the disulfide is formed within a native, high-affinity Fc–FcγR interface, which sterically shields the bond and

limits accessibility to free thiols. Consistent with this expectation, only trace (<1%) higher-molecular-weight species were observed after 3 days in serum, and LC-MS/SDS-PAGE analyses indicate these represent minor self-association rather than albumin exchange, with no loss of functional activity. Importantly, even if minimal thiol exchange were possible, this would not constitute a developability barrier, as such risks are routinely mitigated during standard CMC workflows through formulation optimization and analytical control. We have added this clarification to the manuscript.

- Line 30: „complex forms a 2:1 stoichiometry" – in the case of homodimeric Fc such statements can always be misunderstood, although I realize that this is the usual description – it could be mentioned once that this is one homodimeric Fc with one ligand molecule.

Thank you! We have revised the phrase "complex forms a 2:1 stoichiometry" to the clearer description "one homodimeric Fc with one ligand" in the main manuscript, as suggested.

- Lines 43-33: "functionalize" or "imbue them with novel functionality"- would you consider rewording?

Yes, we have revised the sentence in the main manuscript to: "significant efforts have been made to engineer monoclonal antibodies (mAbs) to enhance their therapeutic properties or equip them with novel functionality".

- Line 92: Numbering scheme for immunoglobulins and the PDB ID should be mentioned at this point.

We have added the immunoglobulin numbering scheme and UniProt identifier to the main manuscript as suggested. The revised sentence now reads: "we identified residues at position 330 in the Fc (following EU numbering on human IgG1 Fc) and 106 in the FcγRIII (UniProt ID P08637) as a potential pair."

- Line 123: Leu 330 –should be Ala or is something wrong with the numbering

Thank you for catching this typo. The residue should indeed be Ala330, and we have corrected the text accordingly. We have also updated the PDB citation in the main manuscript. The revised sentence now reads:

"We initially focused on Leu330 in the Fc and Ile106 in FcγR (PDB: 5D6D)."

- Figure 1: HPLC-SEC in native conditions would be a good addition to this Figure.

HPLC-SEC chromatogram was included in Figure 1 (Fig. 1d), and the main manuscript has been updated accordingly.

- Line 260: "substantially higher than either agent alone" – how many experiments were performed, could the statistical significance be shown?

This dataset was generated from a single experiment, and therefore statistical testing could not be performed; we have clarified this limitation in the manuscript. Importantly, the observed enhancement is consistent with effects we have seen across multiple cell systems. In addition to the data shown here using BT474 and SKOV3 Her2-positive cell lines, we have also evaluated co-administration of T-cell engagers in an independent study using FaDu cells (ATCC HTB-43), which co-express EGFR and Her2. In that system as well, we observed increased tumor-cell killing when combining a primary T-cell engagement signal with a co-stimulatory module.

More broadly, we are continuing to advance this platform, and a systematic exploration of synergy across multiple Fc–FcγR complex constructs and a wider panel of antigen targets and cell lines is currently underway. These studies are substantial in scope and will be reported in a dedicated, detailed manuscript that we intend to submit in the near future. Although these additional datasets fall outside the scope of the present study, early findings across all tested cell lines consistently support the synergistic activity described here.

"For reviewer reference only, we provide an illustrative panel showing this effect in one of these additional cell systems; however, in alignment with the editor's guidance, this figure is now included in the manuscript or Supplementary Information."

[redacted]

- Supplementary Figure 3 cd. In 3c, the experiment has only N=1, and in 3d it is not so clear if the differences between groups could be significant if the deviations were not so large – please could you supplement the data so that these experiments are on the level with the rest of the manuscript.

Supplementary Figure 3c has been updated to include N = 3 biological replicates. Statistical analysis of both Supplementary Figures 3c and 3d showed no significant differences among the αCD3-active groups in either Jurkat or primary T-cell assays. These updates bring the rigor of these experiments in line with the rest of the manuscript. Thank you!

- Line 443:" the detection antibody" – please specify the reagent, including its RRID identifier
As requested, the detection antibodies used in this study are now fully detailed in the Supplementary Information, including RRID identifiers. The main manuscript has been updated to reference these reagents accordingly.

Reviewer #2 (Remarks to the Author):
In this manuscript the authors seek to repurpose the native IgG Fc–FcγR interaction into a universal, covalent docking site to generate multispecific antibodies.

They show that introducing the disulfide between A330C on the Fc and I106C on FcγRIIIa elicits stable, covalent complexes which support addition of payloads such as anti-CD3, anti-CD28, IL-2, and protease-activated IL-2. They then explore the activity of these molecules in vitro and in vivo.

This is a novel and innovative approach which appears to deliver a flexible plug and play technology. The experiments are in the main clear and generate conclusive data. The attraction is the flexibility of the approach and simple modifications that permit this.

However, some areas of the manuscript require clarification and/or improvement.

We sincerely appreciate the reviewer's thorough evaluation and constructive feedback. These comments have greatly improved the clarity and strength of our manuscript. We have carefully addressed each point and incorporated all recommended revisions into the updated version. Our detailed, point-by-point responses are provided below, with our replies highlighted in blue for clarity.

- The introduction states that "we recognized that the FcgR extracellular regions specifically bind to the Fc region of an IgG1 naturally and with relatively high affinity (~1 nM to 1 uM) seemingly unwilling to document that FcgR1 is a high affinity receptor for IgG1 and the others significantly lower.
Why was FcgR1 not selected given the stated aim?

We agree with the reviewer that any of the activating Fcγ receptors, including FcγRI, FcγRIIa, and FcγRIIIa, would be expected to block ADCC when covalently engaged to the Fc, as they all compete for the same FcγR interaction site on IgG1. FcγRI was therefore considered during our initial design efforts.

In preliminary experiments, however, FcγRI fusion constructs exhibited substantially lower expression and reduced assembly efficiency when paired with anti-CD3 or other payloads, whereas FcγRIIIa constructs expressed robustly and consistently formed the intended covalent complexes. Because FcγRI constructs will require further optimization to achieve suitable manufacturability and stability, we elected to focus the present study on FcγRIIIa, where the platform was already performing reliably.

We emphasize that work to extend the platform to FcγRI is ongoing, and our early data suggest that this will be feasible with additional engineering. We have added clarifying text to the manuscript to reflect this rationale while keeping the current work focused on fully validated constructs.

- Given the manuscript reports a new technology with claims of overcoming issues of production etc – statements such as "Both proteins were expressed at high levels and remained stable throughout purification." require substantiation with data.

To address the reviewer's request for substantiation of our statements regarding expression levels and stability, we have now included production yields and native HPLC-SEC chromatograms for all constructs. These data are presented in Figure 1 (Fig. 1d) and Supplementary Fig. 4d–e.

We note that Reviewer #1 raised a similar request, and the same dataset addresses both reviewers' concerns. The corresponding text in the main manuscript has been updated accordingly to reflect these quantitative expression results and biophysical characterization.

- To assess the stability of the complex under physiologically relevant conditions, the authors incubated the purified disulfide-linked complex in PBS and in mouse serum at 37 °C for 7 days. An additional control would be to perform these studies with human serum (the construct is based on human FcgRs and human IgG and so human serum is more relevant).

As requested, we have now included the stability analysis performed in human serum. These data are presented in Supplementary Fig. 1e, and the main manuscript has been updated accordingly to reflect the results.

- The SPR data showing retained binding to FcRn is informative but should be supported with in vitro cell binding studies and/or in vivo assessment of persistence. The authors clearly identify areas for further work (immunogenicity, PK/PD). I agree much of this is outside the remit of this initial paper but think the PK is a fundamental point that should be addressed as it would limit utility of the approach.

We agree that FcRn binding alone is insufficient to establish in vivo persistence and that pharmacokinetic assessment is an essential component of evaluating the translational potential of the platform. To address this, we have included "first-pass" PK data in Supplementary Fig. 4f and incorporated these findings into the main manuscript. These initial studies were designed to provide early confirmation that the Fc–FcγR complexes exhibit measurable in vivo stability and FcRn-dependent persistence.

We emphasize that these PK measurements are not intended as a full PK/PD characterization, which for approved biologics typically includes evaluation of target-mediated drug disposition (TMDD), dose linearity, bioavailability, compartmental modeling, clearance pathways, anti-drug antibody responses, and other determinants of clinical pharmacokinetics. A comprehensive assessment of these parameters is beyond the scope of this initial technology report.

However, we are actively advancing the platform, and expanded PK/PD studies—including TMDD evaluation and multi-dose analysis—are underway as part of our ongoing development efforts. The current preliminary PK data serve as an initial indication of in vivo persistence and address the reviewer's request without broadening the scope of the manuscript beyond its intended focus.

- Some of the terminology used is a little misleading – e.g. "First, the Her2 disulfide complex was generated using FcγRIIIa without the CD3 scFv fusion (αHer2–αCD3mut)." This implies a mutated CD3 scFv not one that is absent – perhaps CD3-null or similar would be more appropriate?

We agree that the original terminology could be misinterpreted as referring to a mutated CD3 scFv. To prevent ambiguity, we have replaced αHer2–αCD3mut with αHer2–αCD3null, which accurately reflects the absence of the CD3-binding domain. This revision has been implemented consistently across the manuscript and figures

- In general there is a lack of benchmarking versus other TCEs for the in vitro and in vivo activity studies.

We thank the reviewer for emphasizing the importance of benchmarking. To directly address this point, we performed a side-by-side in vitro comparison with a clinically approved T-cell engager.

Specifically, we reconstructed the BCMA-targeting T-cell engager teclistamab in an Fc–FcγR disulfide-linked format and compared cytotoxic activity of the two molecules under identical conditions across multiple BCMA-positive cell lines. The Fc–FcγR–based αBCMA–αCD3 complex mediated robust target cell killing with EC_{50} values that overlapped with those of teclistamab (Supplementary Fig. 4g). EC_{50} values across the broader construct panel were also consistent with those reported for approved T-cell engagers (Supplementary Table 2).

We note that clinically approved T-cell engagers represent the outcome of many years of iterative affinity maturation, geometry optimization, in vivo evaluation, and extensive CMC development by commercial teams. The constructs presented here are not positioned as fully optimized drug candidates, but rather as minimal, first-generation implementations of a modular platform architecture. Accordingly, the purpose of this comparison is to demonstrate that the Fc–FcγR format achieves activity within a clinically relevant range, rather than to benchmark against the cumulative effects of long-term product optimization.

While we appreciate the reviewer's comment regarding in vivo benchmarking, in vivo studies were not included in the present work, as we are currently working with physician-scientist collaborators to identify the most appropriate lead application for the platform prior to in vivo optimization, where such resource-intensive studies are most informative. These efforts, including PK/PD and other developability considerations, are ongoing and will be reported separately.

- Figure 1C: there are some extremely broad bands in the SDS PAGE. What are they the result of? This should be commented upon.

The broad bands observed in Figure 1C are most likely due to the heterogeneous N-linked glycosylation of FcγRIIIa, which contains five predicted glycosylation sites. This extensive glycan heterogeneity produces a distribution of molecular weights, resulting in diffuse migration on SDS–PAGE. Similar broad banding patterns are well documented for recombinant FcγRIIIa from commercial sources (e.g., Thermo Fisher A42538 - https://www.thermofisher.com/proteins/product/Human-Fc-gamma-RIIIA-CD16a-176-Phe-His-tag-Recombinant-Protein/A4253). We have updated the figure legend to include this clarification.

- Figure 2 and elsewhere cell experiments are performed with "n=2 donors with triplicates generated " – this is ambiguous as to what is being shown in the figures, with no additional insight in the legend. I presume it is a technical triplicate from a single donor. For robustness and estimation of real biological

variation at least n=3 should be shown with the error bars reflecting the SD from the means of the triplicate values.

We agree that the phrasing "n = 2 donors with triplicates generated" was ambiguous and appreciate the reviewer highlighting this. In the original experiments, EC_{50} values were calculated from technical triplicates, and the standard deviations reflected two independent experiments performed on separate dates using T cells isolated from the same donor's frozen PBMCs. The manuscript has been updated to clearly distinguish technical replicates from biological replicates.

We also acknowledge the reviewer's point that at least n = 3 donors is needed to capture true biological variability. As suggested, we have now expanded the dataset to include cell-killing measurements using T cells isolated from eight independent human donors (Supplementary Fig. 2e). Error bars now reflect biological variation across donors, and the main manuscript has been updated accordingly. We are grateful for the reviewer's comment as these additions bring the experimental rigor of these studies in line with the rest of the manuscript.

- As commented in the discussion, valency is a key feature of IgG and most approved TCES are monovalent. However, 2+1 and other modalities have been developed as the authors describe with Glofitamab. The authors demonstrate nicely with their Her2 biologic the potential of their approach for rapidly generating multivalent molecules.

We appreciate the reviewer's supportive comment and are encouraged that the multivalent capabilities of the Fc–FcγR complex platform were highlighted.

- Minor:
Some typos/errors exist: e.g. on line 91: "identify residues at the interact that"

Thank you! This has been addressed: "identify residues at the interact that" was replaced with "identify residues at the interface that"

RESPONSE TO REVIEWERS' COMMENTS

Reviewer #1 (Remarks to the Author):

In the revised version of the manuscript, the authors have added data on biophysical characterization of the novel constructs, on their pharmacokinetics by determining half-life in mouse studies in vivo, and on the direct toxicity in vivo studies by including data on weight monitoring of experimental animals. Multiple measurements of cell binding data as determined by FACS are also shown for a higher significance of results, and detail level of the Methods section was improved for higher reproducibility. All novel data support author's arguments.
Minor typos:
Line 127: “We initially focused on Leu330 in the Fc“ – this should be Ala330
Line 353: „(approximately 2 hours28)“ – reference 28 not in the correct format
Line 356: reference currently listed as 9 at this point should very probably be 29 (describing PK of glofitamab)

Thank you for your thorough review and thoughtful feedback throughout the evaluation process. We appreciate the time and care you have taken in assessing our manuscript.

Regarding line 127, the statement “We initially focused on Leu330 in the Fc” is correct. To improve clarity, we have revised the sentence to: “We initially focused on Leu330 in the Fc and Ile106 in FcγR from PDB ID: 5D6D.” The subsequent sentence has also been updated to: “The corresponding residues in human IgG1 Fc, Ala330, and the soluble fragment of FcγRIIIa, Ile106, were mutated.”

In addition, we have corrected the other minor typos you identified, including the reference formatting issue on line 353 and the incorrect reference numbering on line 356.

We thank you again for your careful evaluation and constructive suggestions.

Reviewer #2 (Remarks to the Author):

The authors have done an excellent job in addressing my initial comments and have significantly improved the manuscript. i have only 1 remaining issue: the rebuttal letter describes that "We emphasize that work to extend the platform to FcγRI is ongoing, and our early data suggest that this will be feasible with additional engineering. We have added clarifying text to the manuscript" - i coudldnt find any text relating to this

Thank you for your careful review and thoughtful feedback throughout the evaluation process. We appreciate the time and attention you have given to our manuscript.

We apologize for the omission of the clarifying text regarding the extension of the platform to FcγRI. This has now been added to the manuscript and is marked in red for ease of reference.

Thank you again for your careful assessment and helpful comments.
